# Experimental hepatic encephalopathy causes early but sustained glial transcriptional changes

**DOI:** 10.1186/s12974-023-02814-w

**Published:** 2023-05-29

**Authors:** Wouter Claeys, Lien Van Hoecke, Hannah Lernout, Clint De Nolf, Griet Van Imschoot, Elien Van Wonterghem, Daan Verhaege, Jonas Castelein, Anja Geerts, Christophe Van Steenkiste, Roosmarijn E. Vandenbroucke

**Affiliations:** 1 Hepatology Research Unit, Department of Internal Medicine and Paediatrics, Ghent University, 9000 Ghent, Belgium; 2 Liver Research Center Ghent, Ghent University Hospital, Ghent University, 9000 Ghent, Belgium; 3 Barriers in Inflammation, VIB Center for Inflammation Research, VIB, Technologiepark-Zwijnaarde 71, 9052 Ghent, Belgium; 4 Department of Biomedical Molecular Biology, Ghent University, 9000 Ghent, Belgium; 5 IBD Research Unit, Department of Internal Medicine and Paediatrics, Ghent University, 9000 Ghent, Belgium; 6 Department of Internal Medicine and Paediatrics, Ghent University, 9000 Ghent, Belgium; 7 Department of Gastroenterology and Hepatology, Ghent University Hospital, Ghent, Belgium; 8 Department of Gastroenterology and Hepatology, Antwerp University, Antwerp, Belgium; 9 Department of Gastroenterology and Hepatology, Maria Middelares Hospital, Ghent, Belgium

## Abstract

Hepatic encephalopathy (HE) is a common complication of liver cirrhosis, associated with high morbidity and mortality, for which no brain-targeted therapies exist at present. The interplay between hyperammonemia and inflammation is thought to drive HE development. As such, astrocytes, the most important ammonia-metabolizing cells in the brain, and microglia, the main immunomodulatory cells in the brain, have been heavily implicated in HE development. As insight into cellular perturbations driving brain pathology remains largely elusive, we aimed to investigate cell-type specific transcriptomic changes in the HE brain. In the recently established mouse bile duct ligation (BDL) model of HE, we performed RNA-Seq of sorted astrocytes and microglia at 14 and 28 days after induction. This revealed a marked transcriptional response in both cell types which was most pronounced in microglia. In both cell types, pathways related to inflammation and hypoxia, mechanisms commonly implicated in HE, were enriched. Additionally, astrocytes exhibited increased corticoid receptor and oxidative stress signaling, whereas microglial transcriptome changes were linked to immune cell attraction. Accordingly, both monocytes and neutrophils accumulated in the BDL mouse brain. Time-dependent changes were limited in both cell types, suggesting early establishment of a pathological phenotype. While HE is often considered a unique form of encephalopathy, astrocytic and microglial transcriptomes showed significant overlap with previously established gene expression signatures in other neuroinflammatory diseases like septic encephalopathy and stroke, suggesting common pathophysiological mechanisms. Our dataset identifies key molecular mechanisms involved in preclinical HE and provides a valuable resource for development of novel glial-directed therapeutic strategies.

**Graphical Abstract:**

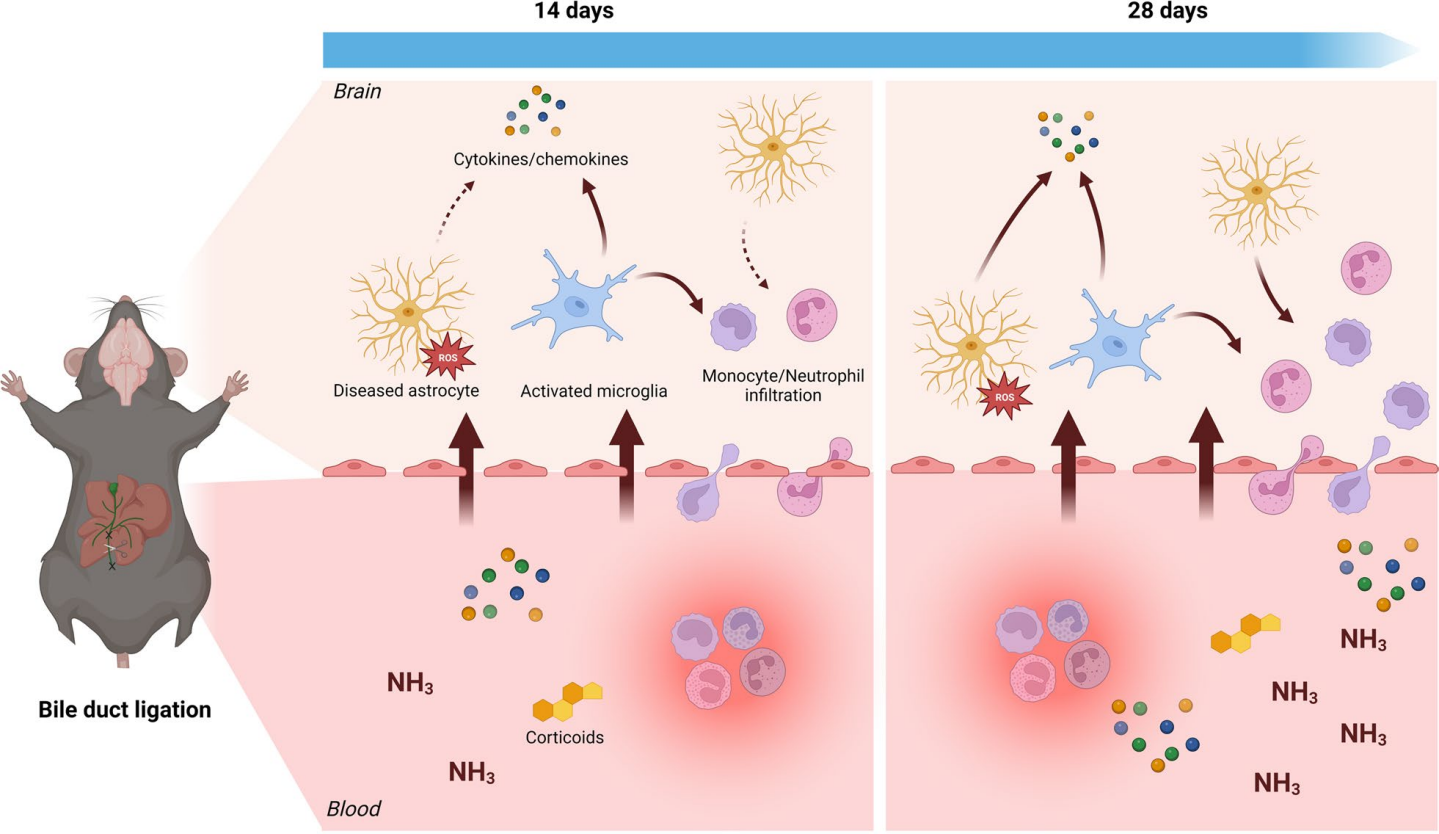

**Supplementary Information:**

The online version contains supplementary material available at 10.1186/s12974-023-02814-w.

## Introduction

Hepatic encephalopathy (HE) is a very common complication of chronic liver disease (CLD). It is estimated that up to 40% of cirrhotic patients experience an episode of overt HE within 5 years after diagnosis [1] while up to 80% of cirrhotic patients suffer from subclinical cognitive impairment, called minimal HE [2]. As HE is a manifestation of severe liver impairment, the prognosis is very poor, with an average mortality of 50% 1 year after onset [3, 4]. Healthcare costs related to HE are ascending and are expected to rise even further as prevalence of non-alcoholic fatty liver disease-associated cirrhosis is also on the rise [5]. While HE is often acute in onset, it is a highly recurrent disorder [3] and can become chronic [1]. Therapeutic options for HE are limited and focus mainly on prevention of overt HE episodes [3, 6]. Moreover, no brain-targeted treatment is available. Importantly, while often considered as reversible, evidence is mounting that even state of the art treatment and liver transplantation cannot fully restore cognitive defects, possibly due to neuronal cell death [7–9] or astrocyte senescence [10]. Clearly, HE is an important healthcare challenge and further investigation into its pathophysiology is warranted to improve outcomes for HE patients.

Hyperammonemia remains the central pillar of HE pathophysiology [2]. Under physiological conditions, ammonia is produced in the gut by the breakdown of dietary nitrogen and is metabolized in the liver through the urea cycle [11]. In cirrhosis, (extra)hepatic ammonia metabolization is insufficient due to a combination of hepatocellular failure, portosystemic shunting, sarcopenia, renal dysfunction and gut dysbiosis, resulting in hyperammonemia. In the brain, ammonia is primarily metabolized by glutamine synthetase in astrocytes, converting ammonia into glutamine while consuming glutamate [12–14]. Current knowledge indicates that the accumulation of glutamine induces cellular swelling and osmotic stress. This, in turn, is compensated by a reduction in other organic osmolytes, such as myo-inositol. The osmotic stress and direct toxicity of ammonia causes oxidative and nitrosative stress. Both osmotic and oxidative/nitrosative stress can worsen each other, leading to protein and RNA modifications, changes in gene expression and cellular senescence [14, 15]. Other factors, with systemic inflammation being key among them, additionally synergize with ammonia to trigger cerebral toxicity [14, 16]. While there has been a significant focus on astrocyte dysfunction in HE research, recent evidence indicates that the condition arises due to the malfunction of multiple cell types within the brain, including endothelial cells and microglia, which received comparatively less attention (reviewed in [15]).

Importantly, the abovementioned evidence is often derived from simplified in vitro model systems precluding strong statements regarding their in vivo relevance. Other reports using crude brain tissue analysis or low-throughput histological evaluation often show limited and conflicting alterations. To illustrate, transcriptome evaluation of brain samples of both HE patients and hyperammonemic models shows relatively little changes compared to control conditions. Moreover, these changes are not consistent between different model systems or even different brain regions within one model system [17–19]. Given the cellular heterogeneity and complexity of the central nervous system (CNS), changes in specific cell types might be masked when assessing crude tissue. Lineage-specific analysis can overcome these limitations, and the value of such cell-type specific evaluation has been shown extensively in many acute and chronic neurological diseases [20–22]. To the best of our knowledge, no such evaluation exists in HE animal models at present.

There are relatively few animal models that recapitulate core HE characteristics in the setting of CLD [23]. We recently evaluated the characteristics of the mouse bile duct ligation (BDL) model, an archetypical model for secondary biliary cirrhosis, observing hyperammonemia and increased cerebrospinal fluid (CSF) glutamine from 14 days after induction [24]. Interestingly, we have shown that both astrocytes and microglia exhibit morphological changes in this model, with microglial arborization decreasing from 14 days after injury and astrocyte arborization significantly increasing 28 days after injury. It is well established that both cell types are critical mediators of the neuroimmune response in acute and chronic brain injury [25]. However, morphological assessment does not reveal all aspects of the underlying cell state, as it is becoming increasingly clear that in different diseases, both morphologically reactive astrocytes [26] and microglia [27] exhibit various underlying molecular states. Importantly, in our previous work, the expression of astrocyte reactivity marker genes was increased before morphological changes were apparent, raising the question whether molecular alterations precede morphological ones [24]. Determination of the underlying cell state could provide insights and possible therapeutic targets for further study in preclinical HE.

Here, we examine the cell-type specific transcriptomic response and functional consequences of both astrocytes and microglia following the BDL model of HE. We adapted recently developed methods to concurrently isolate astrocytes and microglia from adult whole brain tissue samples [22, 28]. Given the apparent differences between kinetics of glial morphological and molecular changes in the BDL model, both early (14 days) and late (28 days) timepoints after BDL were assessed. We observed marked but distinct transcriptional changes in both cell types following BDL induction. This response was more pronounced in microglia and showed limited evolution over time in both cell types. Both cells exhibited enrichment for inflammatory pathways, highlighting the role of neuroinflammation in the BDL model of HE. Interestingly, transcriptomic signatures of both cell types were similar to known signatures in other neurological diseases.

## Materials and methods

### Animals

Unless otherwise stated, 10 to 12-week-old male C57Bl/6j wild type (Janvier Labs, Le Genest-Saint-Isle, France) were used. B6(C)-Ccr2^tm1.1Cln/J^ (CCR2-GFP Tg/+, The Jackson Laboratory 027619) or Ly6g^tm2621(cre)Arte^ (*Ly6g-Cre Tg/*+) crossed with B6.Cg‐*Gt(ROSA)26Sor*^*tm9(CAG‐tdTomato)Hze*^/J (*Rosa26.tdTomato*), generating ‘Catchup^IVM-Red^’ mice [29, 30], were used to visualize infiltrating monocytes and neutrophils in the mouse brain. Mice were housed with 14 to 10 h light and dark cycles and free access to food and water in specific pathogen free conditions. All experiments complied with the current laws of Belgium (Law of 14. August 1986 related to protection and welfare of animals) and EU directive 2010/63/EU, and were approved by the animal ethics committee of Ghent University (EC 2020-051, EC2021-085, EC2022-70 and EC 2023-051). The BDL and sham procedures were performed under sterile conditions as previously described [31]. In short, under isoflurane inhalation anesthesia, a midline abdominal incision was made and the common bile duct was isolated from the flanking portal vein. Next, the common bile duct was occluded with a double ligature of a non-resorbable suture (Mersilk 5-0, Ethicon 682H) and cut in between ligatures to prevent recanalization. Mice received buprenorphine 0.1 mg/kg intraperitoneally for 72 h to prevent postoperative pain and distress [32] and mice were killed 14 and 28 days post-surgery. The study is reported in accordance with ARRIVE guidelines [33].

### Tissue sample collection

Mice were sedated through intraperitoneal injection with an overdose of ketamine (87.5 mg/kg) and xylazine (12.5 mg/kg). After disappearance of paw and tail reflexes, whole blood was isolated using heart puncture, collected in Microvette 500 K2 ethylenediaminetetraacetic acid (EDTA) tubes (Sarstedt, 20.1339.100) and stored at 4 °C until plasma isolation. Afterwards, mice were transcardially perfused using 10 ml 0.2% heparin (Sigma, H‐3125) in ice-cold D‐phosphate-buffered saline (PBS) (Gibco 14190‐094) per mouse (4.50 ml/min). For preparation of single cell suspensions, brains were carefully isolated from the skull and collected in 1.5 ml of ice‐cold 1× Hanks’ balanced salt solution (HBSS)−/− (Gibco, 14175/053) (fluorescence-activated cell sorting (FACS) of astrocytes and microglia) or 1 ml of 1× RPMI 1640 (Gibco, 52400-025) (Immune cell phenotyping). Samples were kept on ice and immediately processed into single cell suspensions. For blood vessel tracing, mice were additionally transcardially perfused with 15 ml (3 ml/min) of ice-cold 5 mg/ml ConcanavalinA-AF594 (Thermo Fisher, C11253) diluted in PBS and 10 ml (3 ml/min) 1% paraformaldehyde (PFA) diluted in PBS. For confocal microscopy analyses, brains were carefully isolated and fixed immediately in 4% PFA for 2 h at 4 °C, followed by 10–20–30% sucrose gradient, embedding in Neg50-OCT cryogel (Prosan, 6502) and storage at − 70 °C.

### Isolation and FACS of astrocytes and microglia from whole brain

For concurrent astrocyte and microglial isolation, the protocol was adapted from previous reports with minor adaptations [28]. In short, brain samples were collected in ice-cold 1× HBSS−/−, the brains were weighed and cut to pieces approximately 1 mm^3^ in size using spring scissors. Brain slurry was dissociated into single cell suspensions using the Neural Tissue Dissociation Kit (P) (Miltenyi Biotec, 130‐092‐628) as performed previously [28] with minor adaptations to the protocol. All steps were performed in laminar flow cabinets and samples were kept on ice unless explicitly stated otherwise. In short, cells were enzymatically dissociated using activated enzyme (P) for 15 min at 37 °C and enzyme (A) for 2 × 10 min at 37 °C under continuous nutation. Additionally, the samples were mechanically dissociated by trituration 10 times with a 5 ml serological pipet in between enzymatic dissociation steps. To stop the enzymatic reaction, samples with diluted with an excess of 1× HBSS−/−. The samples were then passed through a 70 µM cell strainer (BD Falcon, 734‐0003) and mixed with 90% Percoll™ PLUS (equilibrated in HBSS−/−, pH 7.4; Merck, GE17‐5445‐02) and DNase I amplification grade (10 U/µl; Invitrogen, 18068‐015) to obtain a final concentration of 24% Percoll™ PLUS and 75 U/ml DNase. Following, the samples were spun down at 300×*g* for 11 min at room temperature (RT) with a low acceleration and deceleration brake. The myelin layer and supernatant were aspirated and the pellet was resuspended in 50 µl of 0.5% bovine serum albumin (BSA) (Jackson ImmunoResearch, 001‐000‐162) in D‐PBS (Gibco 14190‐094). This process was repeated twice.

Single cell suspensions were pre-incubated with Fc Block (1/100; BD Biosciences, 553142) for 10 min at 4 °C and stained with appropriate antibodies at 4 °C in the dark for 30 min. Antibodies and dilutions are listed in Additional file 6: Table S1. Reactions were stopped by adding an excess of staining buffer, cells were spun down at 400×*g* for 7 min at 4 °C. Pellets were resuspended in FACS buffer and transferred through a 35 µm mesh into a 5 ml Falco®Round‐Bottom Polystyrene Test Tube with Cell Strainer Snap Cap (Fisher Scientific, 08‐771‐23). Cell viability was assessed using DAPI 1/200, added immediately prior to sorting. Flow cytometry and cell sorting was performed on the FACSymphony S6 using the 85 µm nozzle. Cells were sorted into 2 ml Eppendorf tubes containing 450 µl RLT Plus lysis buffer (QIAGEN) containing 1% β-mercaptoethanol. The samples were extensively vortexed and stored at − 80 °C until RNA isolation. Flow cytometry plots were analyzed using FlowJo 10.8.1.

### Immune cell phenotyping of whole brain samples

For immune cell phenotyping, brain halves were collected in 1 ml of ice-cold RPMI 1640 (Gibco, 52400-025) and cut to pieces approximately 1 mm^3^ in size using spring scissors. Brain slurry was enzymatically dissociated as previously described with minor adaptations to the protocol [34]. In short, brain halves were incubated with DNase I, collagenase I and collagenase IV at a final concentration of 30 U/ml, 10 U/ml and 400 U/ml, respectively, for 30 min at 37 °C, with mechanical dissociation using a p1000 micropipette every 10 min. Samples were passed twice through a 70 µM cell strainer (BD Falcon, 734‐0003), centrifuged and supernatant was removed. Cell pellets were resuspended in 10 ml of 30% Percoll™ PLUS (equilibrated in HBSS−/−, pH 7.4; Merck, GE17‐5445‐02) and spun down at 800×*g* for 20 min at 4 °C, with no acceleration and break. Myelin layers and supernatant were removed, and pellets were resuspended in FACS buffer (2 mM EDTA, 2% BSA in 1× HBSS−/−).

Single cell suspensions were pre-incubated with Fc Block (1/100; BD Biosciences, 553142) for 10 min at 4 °C and stained with appropriate antibodies at 4 °C in the dark for 30 min. Antibodies and dilutions are listed in Additional file 6: Table S1. Reactions were stopped by adding an excess of staining buffer, cells were spun down at 400×*g* for 7 min at 4 °C. Pellets were resuspended in FACS buffer and transferred through a 35 µm mesh into a 5 ml Falco®Round‐Bottom Polystyrene Test Tube with Cell Strainer Snap Cap (Fisher Scientific, 08‐771‐23). Flow cytometry was performed on the FACSymphony S6 using the 85 µm nozzle. Flow cytometry plots were analyzed using FlowJo 10.8.1.

### RNA isolation and quality control

RNA from sorted cells was isolated using the RNeasy ®Plus Micro Kit (QIAGEN, 74034) according to the manufacturer’s instructions. The concentration and purity of the RNA was determined using the Agilent RNA 6000 Pico Kit (Agilent 5067-1513) and the Agilent 2100 Bio-Analyzer. A RIN value of 7 or higher was required to proceed. cDNA was synthesized with the SensiFAST™ cDNA Synthesis Kit (Bioline). Real time-qPCR was performed with the Light Cycler 480 system (Roche) using SensiFast SYBR No-Rox (Bio-Line). Volumes were dispensed using the I.DOT (DISPENDIX). Expression levels were normalized to the expression of all stable (at least 2) reference genes, determined using the geNorm Housekeeping Gene Selection Software [35]. Sequences of forward and reverse primers can be found in Additional file 7: Table S2.

### RNA-Seq and downstream analysis

Sequencing was carried out on an Illumina NovaSeq 6000 instrument. Preprocessing of the RNA‐Seq data was performed by Trimmomatic v0.39 [36] and quality control by FastQC v0.11.8 (https://www.bioinformatics.babraham.ac.uk/projects/fastqc/). Mapping to the reference mouse genome was accomplished by STAR v2.7.3a, BAM files were created with Samtools v1.9 and HTSeqCount v0.11.2 was used for counting [37, 38]. The data were split up by cell type, respectively, astrocytes and microglia, and analyzed separately. Limma v3.42.2 was used to normalize both datasets [39]. Principal component analysis (PCA) ellipse analysis was performed and it was determined that four astrocyte samples could be labeled as outliers (outside 2 *z*-scores). These four samples were removed from downstream analysis. Genes which did not meet the requirement of a count per million (CPM) larger than 1 in at least the number of samples equalling the smallest group size, respectively, 4 for astrocytes and 6 for microglia, were filtered out. This resulted in an expression table containing 13,253 genes and 20 samples for the astrocyte dataset and an expression table containing 12,482 genes and 24 samples for the microglia dataset. EdgeR v3.28.1 was utilized to perform differential expression (DE) analysis [40]. Benjamini–Hochberg correction was used to adjust the *p*-values for multiple testing. To be labeled as a differentially expressed gene (DEG), a gene needed to have an adjusted *p*-value < 0.05 and a log_2_ratio > 1 or < − 1. A complete list of DEGs is available in Additional file 8: Table S3. The R package pheatmap v1.0.12 (https://CRAN.R-project.org/package=pheatmap) was used to create a heatmap of the top 25 DEG [according to log_2_ fold change (FC)] between the BDL and Sham group at both 14 days and 28 days for the astrocyte and microglia dataset, respectively. The displayed gene expression is log_2_ normalized. The mean expression value per gene over all samples was calculated and then subtracted from each sample’s particular gene expression value to scale the expression values. The R package EnhancedVolcano v1.13.2 was used to create volcano plots to visualize the results of the DE analyses between the BDL and Sham group at both 14 days and 28 days for the astrocyte and microglia dataset, respectively. The figures plot out the − log_10_ adjusted *p*-value on the *Y*-axis versus the log_2_FC value on the *X*-axis for all genes in the respective expression table. Based on the utilized cut-offs (see above), genes were colored differently: red genes are significant DEGs, blue genes only meet the adjusted *p*-value cut-off, green genes only meet the log_2_FC cut-off and black genes do not meet either requirement. A subset of interesting DEGs was manually chosen and the genes are labeled in the plot.

To be able to compare the expression values of certain genes across datasets, a separate analysis was performed with astrocyte and microglia samples together. The same astrocyte outliers were discarded as in the previous analysis. Instead of calculating log_2_CPM values and performing trimmed mean of *M* values normalization as before, log_2_ transcripts per million (TPM) values were calculated in this combined analysis. This takes into account the length of a gene and facilitates the comparison of expression values between different genes.

Gene Ontology (GO) enrichment analysis was performed using the clusterProfiler R package v3.14.3 [41]. This was conducted on the DEG sets of the DE analyses between the BDL and Sham group at both 14 days and 28 days for the astrocyte and microglia dataset, respectively. The full gene list of the respective expression tables was used as background for the enrichment analysis. All three ontologies (“Biological Pathway”, “Molecular Function”, and “Cellular Compartment”) were included and an adjusted *p*-value cut-off of 0.05 was utilized. All GOs can be found in Additional file 9: Table S4. The top 10 significantly enriched biological pathways were featured in a dot plot for each comparison. These top GO categories were ordered according to geneRatio which is the ratio of the input DEG set annotated in the respective GO term. The adjusted *p*-value is displayed as the color of the dot and the size of the dot is determined by the Count parameter, which is the number of DEGs annotated in the respective GO term. The ClusterProfiler R package v3.14.3 was also used to conduct gene set enrichment analysis (GSEA) using the pre-ranked (according to log_2_FC) full gene distribution of each DE analysis. Various gene sets from literature were collected which represent different microglial and astrocytic transcriptomic profiles from other neuroinflammatory diseases. The enrichment of these gene sets at either end of the full gene distribution of our DE analyses was calculated. The relevant top enriched gene sets are highlighted in GSEA plots. The Running Enrichment Score (RES) is plotted on the *Y*-axis in the top half of the plot for one gene set (green line). The RES was calculated by running down the pre-ranked gene list and updating a running-sum statistic depending on if a gene is in the gene set (increase RES) or not (decrease RES). This score shows whether the gene set is overrepresented at the top or bottom of the pre-ranked list of genes. The barcode lines in the middle of the plot show where the genes in the gene set are situated in the full pre-ranked gene list. The ranked list metric at the bottom of the plot displays how the ranking metric (log_2_FC) evolves as you move down the pre-ranked gene list. It indicates with which phenotype/condition the gene set is correlated. The Normalized Enrichment Score (NES) and adjusted *p*-value for the respective gene sets are shown on the GSEA plot.

Upstream regulator analysis was performed on all DEGs using Ingenuity pathway analysis (IPA) (Qiagen Ingenuity Systems, www.ingenuity.com). Upstream regulators were ranked based on *z*-score. Upstream regulators with |*z*-score| > 2 and *p*-value of overlap < 0.05 were considered significant. The full list of upstream regulators, *z*-scores, *p*-values and target molecules in the dataset can be found in Additional file 10: Table S5.

### Confocal microscopy

Cryosections (14 µm) were cut using the Cryostar NX70, followed by postfixation with 4% PFA for 10 min, washing with PBS and blocking for 1 h at RT with PBS containing 0.1% Triton X-100, 0.5% BSA and 2% serum. Serum was matched to species of secondary antibodies. The same blocking buffer was used to dilute primary and secondary antibodies. Primary antibodies were incubated overnight at 4 °C, followed by washes in PBS and fluoro-conjugated secondary antibody incubation for 2 h at RT. Counterstaining was done with DAPI 1/1000 in PBS. The full list of antibodies can be found in Additional file 11: Table S6. Confocal images were taken with a Zeiss LSM 780 (Zeiss, Germany), using a Plan-Apochromat 25 × 0.8 Imm Korr DIC M27 objective. Image analysis was performed using ImageJ software (version 1.53c, National Institutes of Health).

### Plasma alanine aminotransferase (ALT) measurement

Plasma was prepared from whole blood after centrifugation (10 min, 1300×*g* followed by 15 min, 2400×*g*) and transferring supernatant. Samples were stored at − 80 °C until analysis. Plasma levels of ALT were assessed using the mouse ALT ELISA Kit (Abcam, ab282882) according to the manufacturer’s instructions.

### Statistics

EdgeR v3.28.1 was used to carry out DE analysis on the RNA-Seq data. Benjamini–Hochberg correction was applied to correct the *p*-values for multiple testing. DEGs are genes with an adjusted *p* value < 0.05 and a log_2_ratio > 1 or < − 1.

All data are represented as mean ± standard error of the mean (SEM). Differences were considered significant at *p* < 0.05. For comparison of two groups, unpaired *t*-test or Mann–Whitney test were used based on normality testing of data. For comparison of multiple groups, significance was determined using one-way ANOVA with Dunnett post hoc testing or Kruskal–Wallis with Dunn post hoc testing, based on normality distribution of residuals. All testing was two-sided. Graphpad 9.2 (LaJolla, California) was used for all statistical analyses.

## Results

### FACS of microglia and astrocytes upon BDL

**Fig. 1:**
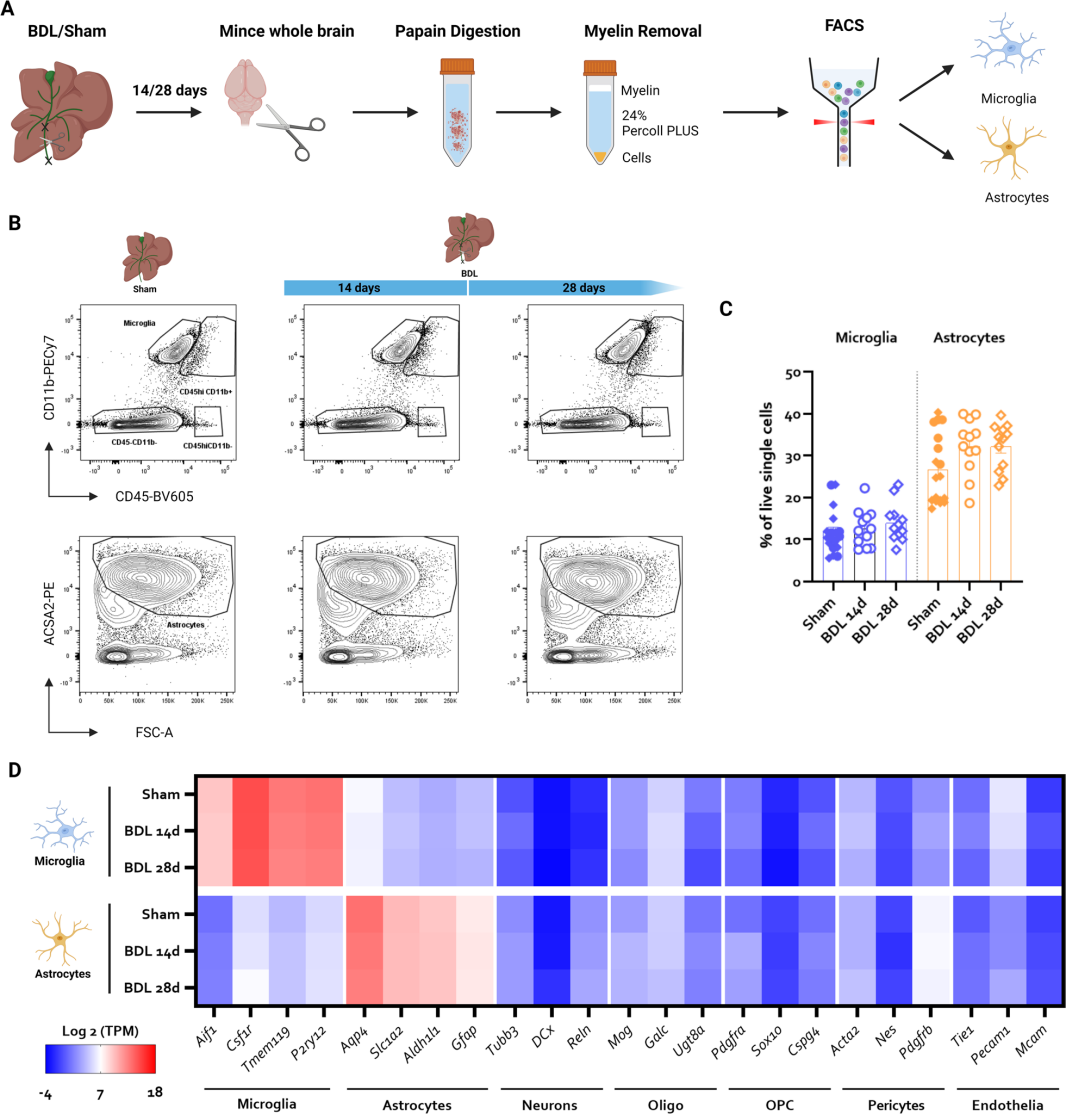
**Isolation of astrocytes and microglia using FACS yields enriched astrocyte and microglial fractions.** **A** Experimental set-up. **B** Representative flow cytometry plots indicating gating strategy used to identify microglia and astrocytes in all mice. **C** Relative abundance of microglia and astrocytes in sham and BDL mouse brains 14 and 28 days after induction. Data are pooled from 2 experiments with *n* = 12–18. **D** Heatmap visualizing log_2_TPM expression levels of cell-type specific markers of microglia, astrocytes, neurons, oligodendrocytes, oligodendrocyte precursor cells, pericytes and endothelial cells in isolated microglia and astrocytes. Heatmaps represent an average of *n* = 4–12 samples per group

To determine gene expression changes in astrocytes and microglia in HE, we sorted astrocytes and microglia, 14 and 28 days after BDL in mice (Fig. 1A). These timepoints were chosen as microglial morphological changes are apparent in BDL mouse brains 14 days after induction, while astrocyte reactivity becomes morphologically apparent 28 days after induction [24]. After exclusion of debris, doublets and dead cells, microglia were defined as CD45^lo-int^CD11b^+^ cells. Astrocytes were defined as CD45^−^CD11b^−^O1^−^ACSA2^hi^ cells, given previous reports that mature oligodendrocytes express low levels of ACSA2 [42], pure ACSA2 based sorting can lead to oligodendrocyte contamination [21] and the ACSA2 antibody can be recognized by Fc Receptors of CD11b^+^ cells (datasheet Miltenyi). ACSA2^int^ cells were excluded based on the fact that this population exhibits a distinct profile on forward scatter—side scatter (Additional file 1: Fig. S1B) and inclusion of this population led to increased expression of neuronal and endothelial markers in the sorted fraction (Additional file 1: Fig. S1C), indicating the presence of contaminating cell types and debris. Detailed gating strategy can be found in Additional file 1: Fig. S1A.

Confirming earlier data [24], no significant differences were detected in the percentage of microglia and astrocytes in BDL mice at either timepoint compared to sham controls, indicating there is no apparent glial cell death nor proliferation (Fig. 1B, C). However, microglial CD45 expression levels were elevated, confirming a reactive status of these cells (Additional file 2: Fig. S2). As expected, astrocytic marker genes (*Aldh1l1*, *Slc1a2*, *Gfap*, *Aqp4*) were highly expressed in sorted astrocytes and only present at very low levels in microglial fractions, while microglial markers (*Aif1*, *Csf1r*, *Tmem119*, *P2ry12*) were highly expressed in sorted microglia and only detectable at very low levels in astrocytes (Fig. 1D). Marker genes of contaminating brain cell types, namely neurons (*Tubb3*, *Dcx*, *Reln*), oligodendrocytes (*Mog*, *Galc*, *Ugt8a*), oligodendrocyte precursor cells (*Pdgfra*, *Sox10*, *Cspg4*), pericytes (*Acta2*, *Nes*, *Pdgfrb*) and endothelial cells (*Tie1*, *Pecam1*, *Mcam*) were only detectable at very low levels in both sorted populations (Fig. 1D). Collectively, this confirms selective enrichment of astrocytes and microglia using the selected strategy.

### Gene expression changes are most pronounced in microglia early after BDL

**Fig. 2:**
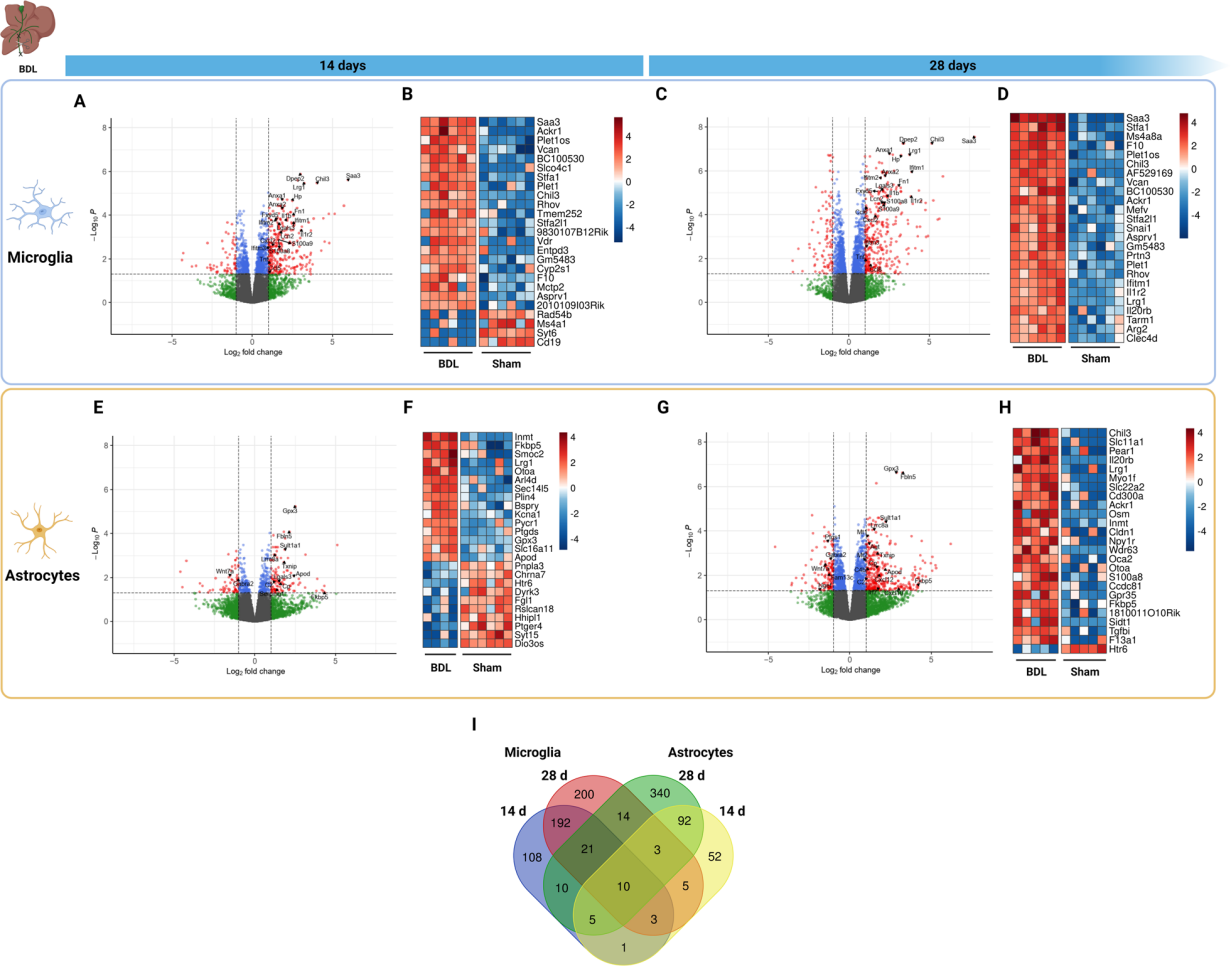
**Glial cells show a distinct but sustained response after BDL compared to sham controls.** **A**–**H** Volcano plots and heatmaps showing microglial (**A**–**D**) and astrocytic (**E**–**H**) gene expression changes 14 days (**A**, **B**, **E**, **F**) and 28 days (**C**, **D**, **G**, **H**) after BDL. In volcano plots (**A**, **C**, **E**, **G**), dotted lines indicate adjusted *p*-value (*y*-axis) and log_2_FC (*x*-axis) cut-offs for differential expression (red: DEG; green: failed *p*-value cut-off; blue: failed |log2FC| cut-off; gray: failed both cut-offs). Selected genes are manually labeled. Heatmaps (**B**, **D**, **F**, **H**) detail the top 25 DEGs ranked on |log2FC|. **I** Venn diagram showing overlapping DEGs based on cell type and time after induction

Next, we performed PCA on both cell types separately to identify whether sham or BDL surgery, as well as the time after induction, has a global effect on transcriptional response. All microglial BDL samples clustered separately from sham microglia. In astrocytes, 4 (*n* = 2 BDL 14d, *n* = 1 sham 28d, *n* = 1 BDL 28d) samples were identified as outliers. As this was not apparent in the corresponding microglial samples and all included mice were both jaundiced at sampling and exhibited increased ALT (Additional file 3: Fig. S3), this was likely not due to failed BDL inductions but rather technical issues during sample processing. Consequently, these samples were therefore removed from further analysis. After outlier removal, all BDL astrocyte samples clustered separately from sham controls. In neither cell type we observed separation on PCA based on time after BDL or sham surgery. Gene expression changes 14 and 28 days after BDL induction were then compared with respective sham controls for microglia and astrocytes separately. DEGs were considered based on the following cut-offs: adjusted *p*-value < 0.05 and |log_2_FC| > 1. In both cell types, a marked change in gene expression was observed, and the number of DEGs increased over time in the BDL model. In microglia, analysis revealed a far greater number of DEGs early in the model. At 14 days, 350 genes (290 up, 60 down) were differentially expressed in BDL microglia compared to sham controls and this number further increased to 448 (355 up, 93 down) 28 days after induction (Fig. 2A–D). In astrocytes, 171 genes (108 up, 64 down) were differentially expressed in BDL mouse brains compared to sham controls after 14 days, and this increased to 495 DEGs (364 up, 131 down) 28 days after induction (Fig. 2E–G). When comparing cell types and timepoints, there was only limited overlap between both cell types (Fig. 2I), indicating a cell-type specific response to BDL. In microglia, we observed a large overlap in DEGs between both timepoints. In astrocytes, while most DEGs are unique to the 28-day group, we again saw a sizeable overlap between both timepoints (Fig. 2I). Furthermore, when comparing timepoints per cell type, no significant differential gene expression between both timepoints could be detected, suggesting limited time-dependent changes in both cell types.

### Validation of microglia and astrocyte-specific differential gene expression following BDL

**Fig. 3:**
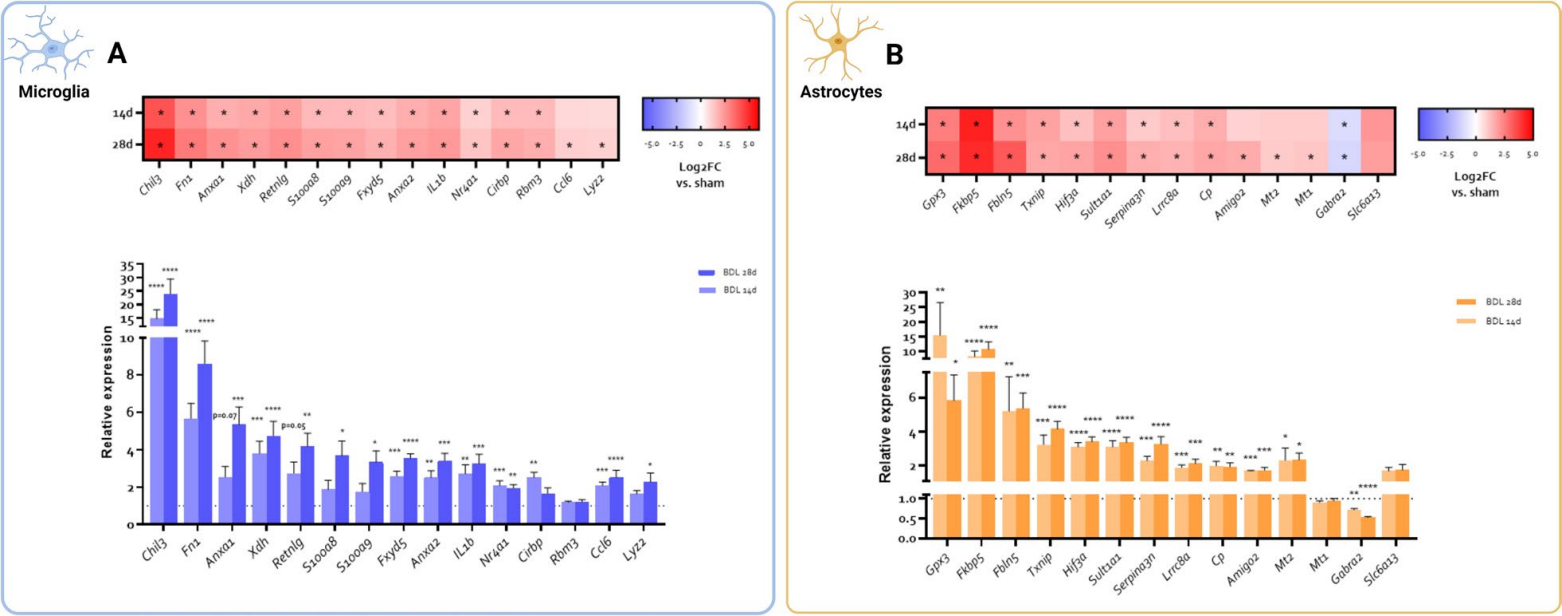
**Validation of gene expression changes in microglia and astrocytes following BDL.** **A** Gene expression of selected genes in isolated microglia in the RNA-Seq experiment (top) and the qPCR validation cohort (bottom). Asterisks denote DE compared to sham controls. **B** Gene expression of selected genes in isolated astrocytes in the RNA-Seq experiment (top) and the qPCR validation cohort (bottom). *p < 0.05, **p < 0.01, ***p < 0.001, ****p < 0.0001

To validate the transcription profiles of the RNA-Seq experiment, a new experiment was performed followed by RNA isolation and qPCR on sorted astrocyte and microglia fractions. In microglia, 13 out of 15 tested genes showed differential expression at both timepoints in the original RNA-Seq experiments (*Chil3*, *Fn1*, *Anxa1*, *Xdh*, *Retnlg*, *S100a8*, *S100a9*, *Fxyd5*, *Anxa2*, *IL1b*, *Nr4a1*, *Cirbp* and *Rbm3*), while 2 genes (*Lyz2*, *Ccl6*) were differentially expressed only at the 28 day timepoint. qPCR confirmed DE of these genes in BDL microglia, with the exception of *Rbm3*, which didn’t show a changed expression level in the validation cohort (Fig. 3A). For astrocytes, 10 out of 14 selected genes (*Gpx3*, *Fkbp5*, *Fbln5*, *Txnip*, *Hif3a*, *Sult1a1*, *Serpina3n*, *Lrrc8a*, *Cp* and *Gabra2*) were differentially expressed at both timepoints in the RNA-Seq experiments, 3 genes were only differentially expressed at 28 days after induction (*Amigo2*, *Mt1*, *Mt2*), and 1 gene (*Slc6a13*) was not differentially expressed at either timepoint. qPCR analysis confirmed DE of selected genes in astrocytes after BDL, apart from *Mt1*, which was not differentially expressed in the validation cohort. Of note, *Amigo2* and *Mt2* expression was already significantly elevated 14 days after BDL, but only in the validation cohort, further emphasizing the limited evolution between both timepoints (Fig. 3B).

### Microglia display altered inflammatory and chemotactic signaling after BDL

**Fig. 4:**
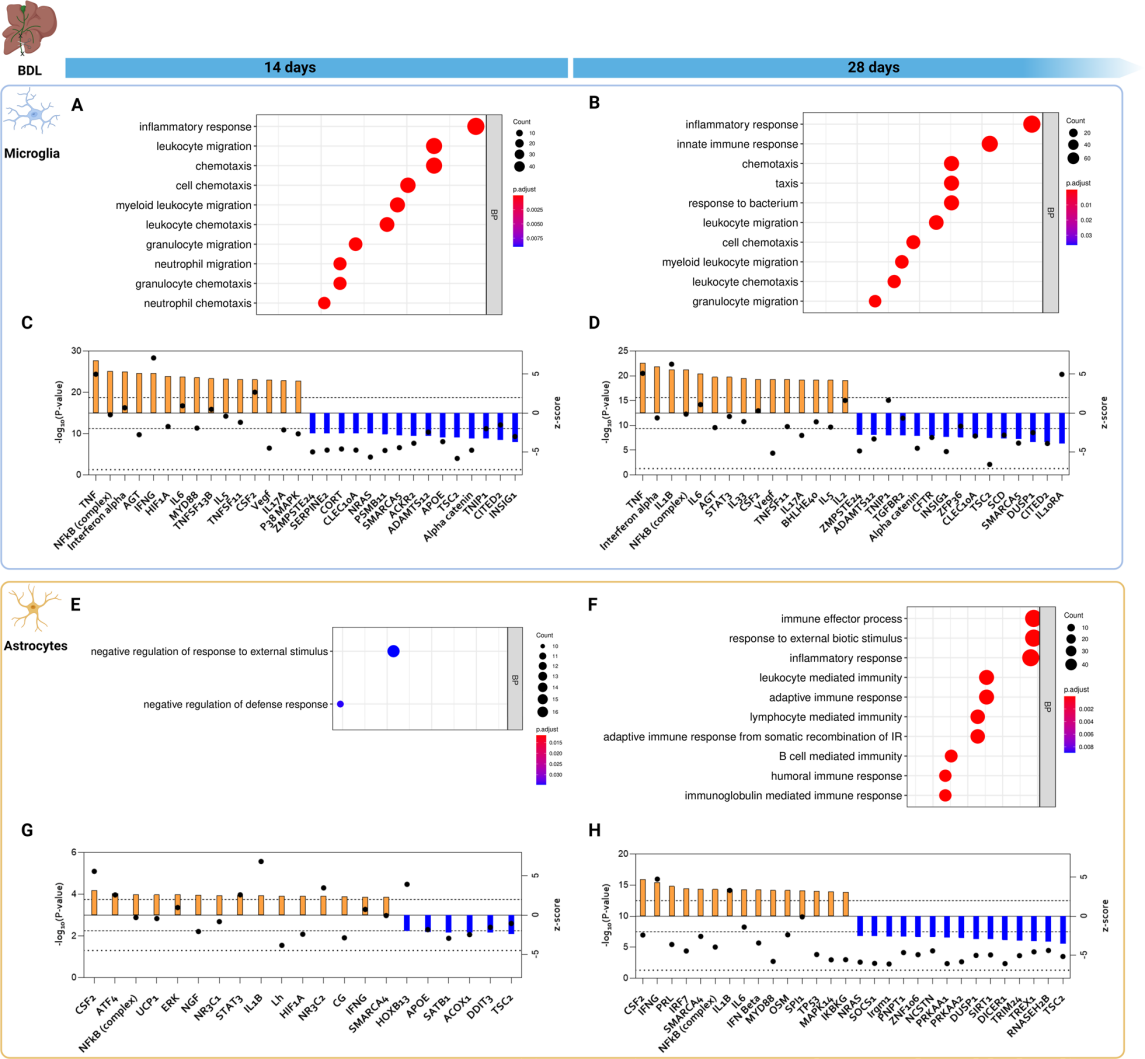
**Glial gene expression is enriched for inflammatory signaling.** **A**, **B**, **E**, **F** Dot plots featuring the top 10 enriched biological pathways in BDL microglia after **(A)** 14 or **(B**) 28 days and astrocytes after **(E)** 14 and (**F)** 28 days. Biological pathways are ordered according to the ratio of the input DEG set annotated in the respective GO terms (geneRatio). Dot color corresponds to the adjusted *p*-value and dot size corresponds to the number of DEGs annotated in the respective GO term. **C**, **D**, **G**, **H** IPA analysis with identified upstream regulators of microglial signaling 14 (**C**) and 28 days (**D**) after BDL induction and of astrocytic signaling 14 (**G**) and 28 days (**H**) after BDL induction. Upstream regulators are ranked based on *z*-score. Bars represent *z*-score (orange: activation; blue: inhibition) and dots represent − log10 (*P*-value). Dotted lines indicate the *p*-value cut-off (*p* < 0.05) and dashed lines indicate the *z*-score cut-off (|*z*-score| > 2)

To elucidate the underlying function of differentially expressed genes in both cell types, we performed GO analysis on the DEG set for astrocytes and microglia at both the 14 and 28 day timepoint. For microglia, the top enriched biological pathway was ‘inflammatory response’ 14 days after induction. Furthermore, ‘chemotaxis’, ‘cell chemotaxis’, ‘leukocyte chemotaxis’, and ‘leukocyte migration’ pathways were significantly enriched (Fig. 4A). Underlying these pathways, we detected differential expression of cytokines (*Il1b*, *Tnf*, *S100a8*, *S100a9*), acute phase response signaling genes (*Lcn2*, *Saa3*), chemokines (*Ccl5*, *Cxcl2*, *Ccl17*, *Ccl22*), anti-inflammatory markers (*Chil3*, *Anxa1*, *Anxa2*) and cell adhesion molecules (*Itgal*, *Sell*) (Additional file 9: Table S4). At 28 days, a very similar pattern was observed, with the ‘inflammatory response’ signaling pathway being the most affected biological pathway. Additionally, there was significant enrichment of ‘chemotaxis’, ‘cell chemotaxis’, and ‘leukocyte chemotaxis’ (Fig. 4B). Apart from the genes mentioned earlier, we also observed differential expression of monocyte chemoattractants *Ccl6* and *Ccl8* at this specific timepoint (Additional file 9: Table S4).

To assess which factors drive these transcriptomic changes, an upstream regulator analysis using IPA was conducted. At the 14 day timepoint, tumor necrosis factor (TNF) was the upstream regulator with the highest *z*-score, with increased expression of pro-inflammatory cytokines (*Il1b*, *Tnf*), TNF response elements (*Tnfaip*, *Tnfsf13*, *Tnfsf14*) and chemokines (*Ccl5*, *Ccl17*, *Ccl22*, *Cxcl3)*. Furthermore, IFN-γ was the most significant upstream regulator, reflected by differential expression of several interferon response genes such as *Ifi16*, *Ifi47*, *Ifitm1*, *Ifitm2*, *Ifitm3*, *Lgals3* (Fig. 4C; Additional file 10: Table S5). A very similar pattern is seen 28 days after induction, with TNF being the upstream regulator with the highest *z*-score. Further down the list, we observed pro-inflammatory cytokines (IL6, IL1b) and pro-inflammatory mediators (NFκB, MYD88), as well as factors involved in hypoxia and angiogenesis (VEGF, AGT at both timepoints, and HIF1A uniquely at 14 days after induction) (Fig. 4C, D; Additional file 10: Table S5).

### Astrocytes exhibit alterations in inflammatory, hypoxia and corticoid response signaling upon BDL

In contrast to the pronounced early changes in microglia, GO analysis of the astrocytic transcriptome at 14 days revealed a limited gene set enrichment with two significantly affected biological pathways, ‘negative regulation of response to external stimulus’ and ‘negative regulation of defense response’ (Fig. 4E), with DE of oxidative stress response elements like *Apod* [43] and *Pxdn* [44], as well as the senescence marker *Cdkn1a*. Interestingly, *Wnt7a* levels were decreased, which has recently been linked to blood–brain barrier (BBB) disruption (Additional file 9: Table S4) [45]. At 28 days however, we did see significant enrichment of several biological pathways, mainly related to immune function and inflammatory signaling. The top enriched biological pathways were ‘immune effector process’, ‘response to external biotic stimulus’ and ‘inflammatory response’ (Fig. 4F). This included upregulation of monocyte and neutrophil chemoattractants (*Cxcl10*, *Cxcl12*, *Ccl6*), complement factors (*C4*, *C1qb*, *C2*, *Cfh*) and MHC Class I related genes (*H2-DMA*, *H2-T23*, *H2-M3*, *H2-Ab1*) (Additional file 9: Table S4). This suggests that astrocytes adopt a pro-inflammatory phenotype at 28 days after induction.

To assess which factors drive these transcriptomic changes, an upstream regulator analysis using IPA was conducted. While *p*-values are much lower in the 28-day analysis, again overlap was found between the two timepoints, suggesting conserved changes. CSF2 (encoding GM-CSF) was identified as the top upstream regulator. Corticoid receptor signaling (NR3C1 (glucocorticoid receptor), NR3C2 (mineralocorticoid receptor)) was identified as a driver of astrocyte transcriptional changes, with upregulation of corticoid response elements (*Sgk1*, *Tsc22d3*, *Fkbp5*), most pronounced 14 days after injury. Furthermore, we identified the activation of hypoxia-related signaling via HIF1A, reflected by the induction of genes related to hypoxia and oxidative stress response (e.g., *Hif3a*, *Gpx3*, *Ndrg1*, *Txnip*) and cell cycle regulatory genes (e.g*., Cdkn1a*, *TP53rk*) (Fig. 4G; Additional file 10: Table S5). At the 28-day timepoint, CSF2 was again the upstream regulator with the highest *z*-score. Similar to microglia, when ranking on adjusted p-value, IFN-γ emerged as the top driver of transcription in these cells, with altered transcription of interferon response elements *Ifit1b*, *Irf7*, *Irf8* and *Ifitm3*. Additionally, we observed inflammatory mediators like IL1B, IL6, TNF, NFκB and MYD88 as possible transcriptional regulators, concomitant with increased expression of chemokines (*Cxcl10*, *Cxcl12*) and complement factors (*C4*, *C1qb*, *C2*, *Cfh*). Finally, TP53-derived signaling was found to be activated, along with increased expression of *Cdkn1a* and *Gadd45b*, suggesting a possible role for astrocyte senescence (Fig. 4H; Additional file 10: Table S5). In summary, pathway analysis of the astrocyte transcriptome following BDL suggests alterations in inflammatory signaling, interferon response, hypoxia/oxidative stress, cellular senescence and (gluco)corticoid signaling.

### Ly6C^hi^ monocytes and Ly6G^hi^ neutrophils accumulate progressively in the BDL mouse brain

**Fig. 5:**
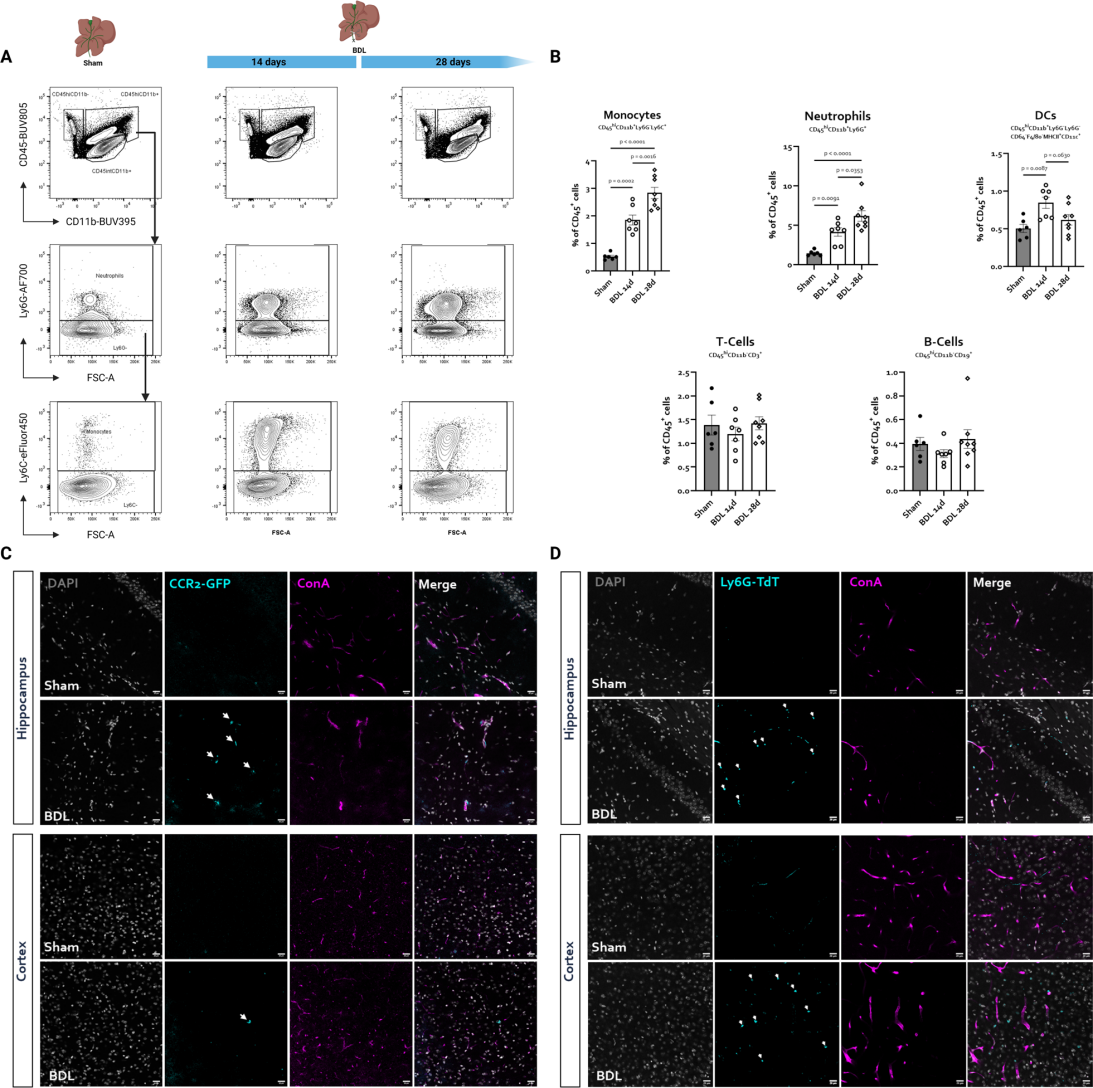
**Immune phenotyping reveals progressive influx of monocytes and neutrophils in BDL mouse brains.** **A** Representative flow cytometry plots indicating gating strategy used to identify monocytes and neutrophils. **B** Relative abundance of neutrophils, monocytes, T cells, B cells and DCs in sham/BDL mouse brains 14 and 28 days after induction. Data are derived from a single experiment with *n* = 6–8/group. Representative images showing **C** CCR2-positive monocytes, **D** Ly6G-positive neutrophils and concanavalin A positive blood vessels in cortex and hippocampus of sham and BDL mice, 14 days after surgery. Scale bar represents 20 µm

As the microglial transcriptome hinted towards attraction of immune cells (Fig. 4A, B), we assessed whether we were able to observe increases in myeloid (CD45^hi^CD11b^+^) or lymphoid (CD45^hi^CD11b^−^) cells in our RNA-Seq experiments. Confirming earlier research [46], no differences in relative amount of lymphoid cells in the brain at either timepoint after BDL could be detected. On the other hand, a stepwise increase in brain-infiltrating myeloid cells over time was apparent (Additional file 4: Fig. S4). We previously observed that papain-based brain tissue digestion results in partial or full degradation of key epitopes used in immune phenotyping, similar to what is reported elsewhere [47]. Therefore, detailed immune phenotyping experiment were performed using collagenase based digestion as described in other reports [34]. The full gating strategy can be found in Additional file 5: Fig. S5. Confirming earlier reports [48], we observed an influx of CD45^hi^CD11b^+^Ly6G^−^Ly6C^hi^ monocytes into the brains of BDL mice (Fig. 5A, B). Interestingly, this influx exhibited a stepwise increase over time, suggesting continued attraction of monocytes during disease progression. We additionally detected a stepwise increase in CD45^hi^CD11b^+^Ly6G^hi^ neutrophils in BDL mouse brains, with neutrophils making up more than 5% of all CD45^+^ cells 28 days after induction (Fig. 5A, B). In contrast, levels of CNS CD3^+^ T cells, CD19^+^ B cells were not changed at either timepoint. Levels of dendritic cells were transiently elevated 14 days after injury, however these changes were small and not sustained (Fig. 5A, B). To assess whether monocytes and neutrophils attracted to the brain penetrate through the BBB, we performed BDL in mice expressing green fluorescent protein (GFP) in C–C chemokine receptor type 2 (CCR2) positive inflammatory monocytes and mice expressing TdTomato (TdT) in Lymphocyte antigen 6 complex locus G6D (Ly6G) positive neutrophils. Transcardial perfusion of fluorescently labeled concanavalin A was performed to label blood vessel lumens. CCR2-positive cells, although rare, were only detectable in BDL mouse brains, both in the hippocampus and cortex. CCR2-positive cells were apparent both adherent to blood vessels and in the brain parenchyma near blood vessels, suggesting transendothelial migration (Fig. 5C). Similarly, Ly6G-positive cells were uniquely found in BDL cortex and hippocampus, both in the vicinity of blood vessels and deeper in the CNS parenchyma (Fig. 5D).

### Glial transcriptional profiles in BDL exhibit similarities with acute and chronic neuroinflammatory disorders

**Fig. 6:**
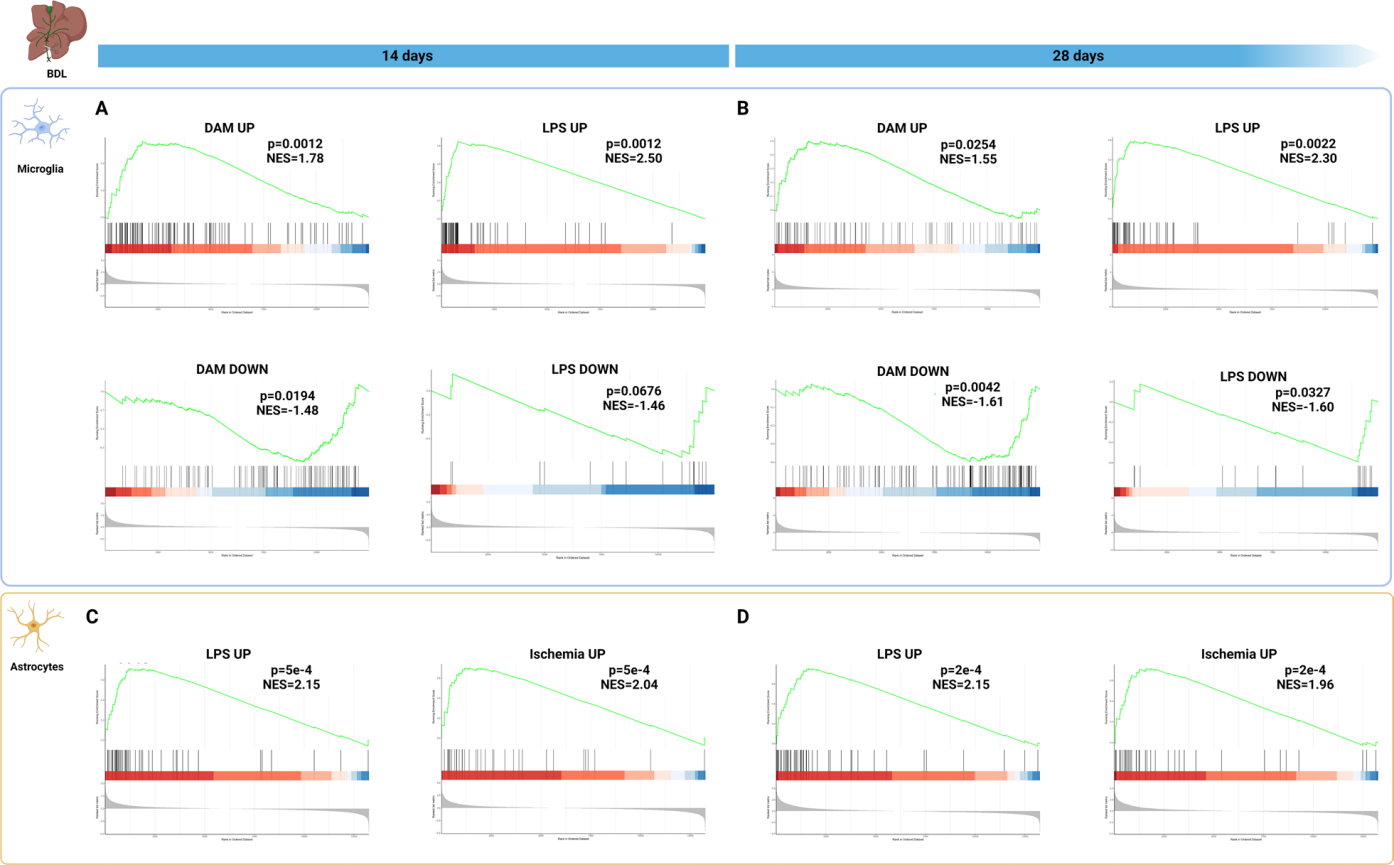
**Gene set enrichment analysis reveals significant overlap between the glial transcriptome in BDL mice and neuroinflammatory disorders.** Comparison of BDL microglia (at **A** 14 and **B** 28 days after induction) and astrocytes (at **C** 14 and **D** 28 days after induction) with published gene signatures. GSEA plots include the RES, NES, adjusted *p*-value, placement of the member genes and ranked list metric plot for that full gene distribution

In recent years, much progress has been made with regard to uncovering transcriptomic signatures of astrocytes and microglia in both health and disease. With regard to microglia, specific gene expression patterns in response to acute and chronic damage have been identified. The most well-known of these is called disease-associated microglia (DAM), which was originally described in models of Alzheimer’s disease (AD) and has since been extended to other neurodegenerative diseases like amyotrophic lateral sclerosis [49]. Per GSEA, presence of various gene sets from literature was evaluated in our DEG list (ranked based on log_2_FC), identifying whether these selected gene sets are significantly enriched in our dataset and whether enrichment occurs at the top or the bottom of the ranked DEG list (NES). When comparing microglia in our dataset with the DAM gene expression pattern, we were able to find significant overlap between both patterns at the two timepoints (upregulated genes: *p* = 0.0012, NES = 1.78 at 14 days; *p* = 0.0254, NES = 1.55 at 28 days; downregulated genes: *p* = 0.0194, NES − 1.48 at 14 days; *p* = 0.0042, NES = − 1.61 at 28 days) (Fig. 6A, B). As HE is often acute in onset and precipitated by infection, we additionally compared our dataset with a report on microglial changes in acute lipopolysaccharide (LPS)-challenged mice [50]. Again significant overlap between both datasets could be found (upregulated genes: *p* = 0.0012, NES = 2.50 at 14 days; *p* = 0.0022, NES = 2.30 at 28 days; downregulated genes: *p* = 0.0676, NES = − 1.46 at 14 days; *p* = 0.0327, NES = − 1.60 at 28 days) (Fig. 6A, B).

In astrocytes, gene expression following LPS-induced neuroinflammation and ischemic stroke were found to be highly different, leading to the description of two distinct astrocyte expression patterns, namely A1 (LPS-induced) and A2 (middle cerebral artery occlusion (MCAO)-induced) astrocytes [51]. Other reports have highlighted that astrocyte responses are often disease-specific and do not necessarily follow the A1–A2 dichotomy [20, 52]. To avoid oversimplification in A1 and A2 expression patterns, we will refer to LPS-induced and ischemia-induced astrocytic phenotypes. Given the enrichment of inflammatory signaling and hypoxia-related signaling, comparison with these subsets could prove interesting. Using GSEA, we were able to identify significant overlap of astrocytes following BDL with gene expression patterns of both LPS-induced and ischemia-induced astrocytes at both timepoints (LPS-induced: *p* = 0.0005, NES = 2.15 at 14 days; *p* = 0.0002, NES = 2.15 at 28 days; ischemia-induced: *p* = 0.0005, NES = 2.04 at 14 days; *p* = 0.0002, NES = 1.96 at 28 days) (Fig. 6C, D). Indeed, when assessing the putative list of marker genes generated by Liddelow et al. [53], pan-reactive astrocyte markers *Cp* and *Serpina3n*, as well as LPS-specific markers *H2-T23*, *Serping1*, *H2-D1*, *Fbln5*, *Fkbp5* and *Psmb8* were already differentially expressed 14 days after injury. At 28 days, we additionally observed increased expression of the pan-reactive marker *Cxcl10* and the LPS-specific marker *Amigo2*. Collectively, these data suggest that microglial and astrocytic gene expression phenotypes in experimental HE resemble those in other neuroinflammatory disorders despite the unique features of hyperammonemia and cholestasis.

## Discussion

Astrocytes are often considered to be the main affected cell type in HE. However, multiple lines of evidence have shown a more complex picture, with e.g., an important role for microglia in (pre)clinical HE [15, 24, 54]. We have previously established microglial and astrocyte morphological changes in the BDL model of experimental HE [24]. However, the molecular mechanisms underlying these morphological changes remain largely unknown. Given the high prevalence, morbidity, mortality and the lack of targeted therapy in HE, it is imperative to further elucidate microglial and astrocyte dysfunction in this disorder. In this study, we performed RNA-Seq of whole-brain isolated microglia and astrocytes at two timepoints in the BDL mouse model to assess model- and time-dependent transcriptional responses of these cells in experimental HE. We show a sustained response in both cell types, more pronounced in microglia, and with relatively limited evolution in between both timepoints. Transcriptomic changes were distinct in astrocytes and microglia, but enrichment for inflammatory signaling was found in both cell types. Overlapping transcriptional response with other neuroinflammatory disorders was observed, highlighting that mechanisms conserved across neuroinflammatory disorders could also play a role in HE.

Surprisingly, microglial transcriptional changes were more pronounced than astrocytic changes in the BDL mouse model. This suggests that microglia, and not only astrocytes, play an important role in HE development from disease onset. We additionally saw a large overlap in between both timepoints, indicating only small changes to an established pathogenic phenotype between 14 and 28 days following BDL. In line with patient data [18], we found increased microglial expression of both pro-inflammatory (e.g., *Tnf*, *Il1b*, *Ccl5*, *Ccl6*, *Ccl8*, *S100a8*, *S100a9*) and anti-inflammatory (*Chil3*, *Anxa1*, *Anxa2*, *Ccl17*, *Ccl22*) genes [55–58]. Therefore, microglia might exert both detrimental and protective effects, depending on the spatial and temporal context. Future studies identifying and modulating microglial subsets and their (spatial) heterogeneity will be able to unravel these differential roles.

Besides inflammation, microglial signaling was significantly enriched for chemotactic pathways, with increased expression of monocyte (e.g., *Ccl6*, *Cxcl3*) and neutrophil (*Ccl5*) chemoattractants. As we were able to show a progressive influx of inflammatory monocytes and neutrophils into BDL mouse brains, this suggests that microglia play a role in attraction of innate immune cells to the HE brain, as previously suggested by another group [48]. While monocyte attraction to the BDL mouse brain has been previously reported [46, 48], the infiltration of neutrophils is a novel finding.

At present, it is unclear whether these infiltrating myeloid cells exert protective or detrimental functions in HE. As evident from a recent review, monocyte function in other acute and chronic neurological disorders is varied, but generally associated with increased tissue damage [59]. In line with this, previous research has suggested that blocking monocyte influx into the brain attenuates CNS injury in BDL mice [48, 60]. However, many of the model systems—e.g., full body CCR2 KO mice and platelet depletion paradigms—that were used in these studies, have been shown to significantly attenuate liver injury in BDL mice [61, 62], and results should therefore be interpreted with caution. Blocking microglial activation pathways or monocyte attraction signaling specifically from the brain might provide a clearer picture into the role of infiltrating monocytes and monocyte–microglia interaction in experimental HE.

In contrast to monocytes, the role of neutrophils in CNS inflammation is unclear. Neutrophils have often been regarded as important but short-lived cells in the acute first line defence against pathogens. However, evidence is mounting that neutrophil function is (1) more complex than simple phagocytosis of pathogens/debris and (2) is non-redundant in more long-term neuroinflammatory disorders [63, 64]. The continued attraction of neutrophils to the BDL mouse brain equally suggests a role in the continued brain injury. Neutrophils can induce BBB damage and increase vascular permeability in neuroinflammation through release of proteinases [64] and cerebral hypoperfusion through formation of plugs [64], two mechanisms implicated in experimental and clinical HE [2, 24, 65]. Microglia can attract neutrophils to aid in pathogen and debris removal [64] and neutrophils can again leave the CNS after interaction with microglia, possibly influencing the peripheral immune response [66]. Astrocyte–neutrophil interactions function as a positive feedback loop, aggravating the cytokine response upon injury [64]. Whether any of these mechanisms occur in liver failure-induced brain injury remains to be determined.

It is unlikely that microglia are the only source of chemoattractant signals from the BDL brain. Indeed, astrocytes upregulated expression of monocyte chemoattractants like *Cxcl10* and *Cxcl12*. Moreover, it has previously been suggested that monocytes are attracted to the BDL brain in a CCL2 dependent manner [48], and we have indeed shown increased brain CCL2 in BDL mice in our previous work [24]. However, we were unable to show increased *Ccl2* expression in microglia after BDL, suggesting that the presence of alternative sources of chemokines like neurons (as has been shown in acute liver failure [67]), might additionally play a role in innate immune cell attraction to the brain.

Similar to microglia, we observed an early and sustained astrocytic transcriptional response to BDL. While GO analysis revealed limited enrichment at the 14-day timepoint, DEG overlap between both timepoints was substantial and we observed no differential gene expression between both timepoints. Moreover, BDL-induced astrocytes showed overlapping gene expression with LPS and ischemia-induced phenotypes already 14 days following BDL. While in our previous report, morphological changes in glial fibrillary acidic protein (GFAP) positive astrocytes only became apparent later in the model, expression levels of reactivity markers like *Fkbp5* and *Cp* were detectable before these morphological changes with limited evolution over time [24]. Collectively, these findings indicate that evolution in the astrocyte transcriptional response is limited in the BDL model after 14 days. The limited amount of DEGs and pathway enrichment at 14 days might be attributable to a lower sample size and variability in the model system.

Differential gene expression in astrocytes was enriched for inflammatory signaling as it was in microglia, with increased expression of chemokines (e.g., *Cxcl10*), complement factor elements (e.g., *C4b*) and known LPS-responsive genes (e.g., *Serpina3n*). Interestingly, GM-CSF, the top identified regulator of astrocytic transcription has recently been found to boost development of pathogenic, pro-inflammatory astrocytes in a mouse model of multiple sclerosis [68].

Given that ammonia is a relatively astrocyte-specific toxin [12], it has been proposed to label astrocytes in HE as ‘diseased’ (i.e., affected by a cell-autonomous disturbance) rather than ‘reactive (i.e., secondary to an extrinsic signal like LPS) [26]. Astrocytes, however, can obtain ‘reactive’ characteristics secondary to this ‘diseased’ state. We have observed altered GFAP morphology in BDL mouse astrocytes [24], DE of classical reactivity markers (e.g., *Cp*, *Amigo2*, *Fbln5*, *Psmb8*), overlap with known phenotypes of reactive astrocytes and functional BBB disturbances with associated *Wnt7a* downregulation [45], all indicative of astrocyte ‘reactivity’. However, other well-known and oft-used reactivity markers like *Gfap*, *Lcn2* and *C3* were not differentially expressed in our dataset (additionally suggesting altered GFAP positive immunoreactivity is potentially posttranslational in nature), indicative of key differences with classical ‘reactive astrocytes’. Therefore, our data suggest a ‘diseased’ astrocytic phenotype with arising reactivity characteristics in BDL mice.

While inflammatory signaling accounts for many findings in both astrocytes and microglia, some additional findings in this study warrant further research. Firstly, both in astrocytes and microglia, HIF1A was found to drive both astrocytic and microglial transcription, with increased transcription of hypoxia-responsive elements like *Ddit4* and *S100a8* [69] in microglia and *Hif3a* and *Cdkn1a* in astrocytes. This suggests a role for cerebral hypoxia in astrocyte and microglial dysfunction in BDL mice, consistent with a decreased cerebral blood flow in clinical HE [70, 71], as well as increased brain susceptibility to hypotension [72] and decreased cerebral oxygen pressure in BDL rats [65]. Suggested underlying mechanisms include a direct effect of ammonia on brain oxygen homeostasis [65], an ammonia-induced dysfunction of cerebral blood flow autoregulation [73] or a decreased energy demand from the brain secondary to ammonia-induced increases in GABA-ergic tone [74]. Of interest, increased expression of oxidative stress response genes (e.g., *Gpx3*, *Ndrg1*, *Txnip*, *Mt1*, *Mt2*) was found in astrocytes. While the presence of CNS oxidative stress in HE remains controversial [2] and we previously reported unaltered levels of oxidative stress markers in the CSF of BDL mice [24], ammonia is well known to induce oxidative stress in vitro [75, 76] and in vivo [76], suggesting our earlier findings might have been masked by crude CSF analysis. Mechanistically, ammonia has been shown to induce cellular senescence in an oxidative stress dependent manner in vitro [77] and in vivo [78]. Astrocyte senescence has been put forward as a cause of irreversible cognitive deterioration despite state of the art treatment of HE [10]. In agreement with this, we found increased astrocyte expression of the well-known senescence marker *Cdkn1a* and TP53 was identified as a possible driver of astrocyte transcriptomic changes, both indicating in vivo astrocyte senescence following BDL.

Secondly, corticoid response signaling was found to be enriched in astrocytes, most pronounced 14 days after injury, with upregulation of known astrocytic (gluco)corticoid receptor signaling response elements (*Sgk1*, *Tsc22d3*, *Fkbp5*) [79, 80]. Astrocytes have recently been identified as the prime responders to glucocorticoids in the brain [81] and glucocorticoid signaling is needed for astrocytic glucose uptake and memory formation [79]. Chronic stress paradigms induce astrocytic glucocorticoid resistance however, resulting in decreased astrocytic ATP release and depressive-like behaviors [82]. Interestingly, bile acid-mediated steroidogenesis and increased circulating corticosterone levels have recently been reported in BDL mice [83] and cholestatic patients exhibit increased cortisol levels correlating with disease severity [84]. On the other hand, in a model of HE in subacute liver failure, glucocorticoid receptor signaling inhibition rescued motor deficits [85]. Therefore, whether increased astrocytic (gluco)corticoid-mediated signaling is secondary to cholestasis or rather a universal feature of liver failure and HE remains a subject for further investigation.

Finally, besides upregulation of *Lrrc8a*, critical to volume regulation upon cell swelling, we found limited evidence for altered osmotic or mechanosensory stress pathway signaling [86]. Increased glutamine and compensatory osmolyte changes are well-established in (pre)clinical HE and in our model [24, 87, 88]. However, the presence and pathophysiological importance of (low-grade) cerebral edema in HE associated with CLD, as well as its relation to astrocyte swelling remains controversial [89, 90]. In accordance with data in BDL rats, the absence of significant osmotic stress seems to suggest near complete compensation of the glutamine rise by depletion of the osmolyte pool [87]. Changes in GFAP immunoreactivity are therefore likely related to reactivity rather than manifest swelling [24]. Whether changes in osmolyte composition affect the ability to cope with additional volume challenges (e.g., acute ammonia or endotoxin challenge), as is the case in BDL rats, remains to be investigated [91, 92].

This study has several limitations. First off, the BDL model is characterized by extensive cholestasis, which in itself can affect the brain [93]. In this study, no early timepoint was included to evaluate pure cholestasis compared to later established advanced CLD. However, in the current landscape of available HE models, it is very difficult to disentangle the relative contribution of cholestasis, advanced CLD and hyperammonemia. Investigations similar to ours in models of pure hyperammonemia, cholestasis without liver function failure and advanced CLD without cholestasis might shed light on the role and the relative contributions of each of these factors to the observed phenotype.

Both for astrocytes and microglia, significant functional and spatial heterogeneity has been observed in health and disease [42, 94, 95], with changes often occurring in (spatially restricted) subsets of glial cells, which cannot be assessed using the presented methods. As hyperammonemia and HE are well-known to differentially affect brain regions [14, 17], investigation of (spatial) heterogeneity and evaluation of small subsets of cells are an obvious point of further research. In line with our data showing infiltrating immune cells in both cortex and hippocampus, previous reports show glial dysfunction in the hippocampus, cortex and cerebellum of HE animals [9, 24, 96]. Therefore, studies utilizing single-cell RNA-Seq and high-resolution spatial transcriptomics approaches, focusing on these brain regions, will be able to give us insight into cellular and spatial diverseness in HE.

Finally, some of the genes differentially expressed in microglial samples after BDL like *Hp* and *Sell* have been proposed as differentiating markers between microglia and peripheral myeloid cells [97], and levels of the monocyte marker *Ly6c2* (only present in low amounts) were increased after BDL. Indeed, we and others have shown CNS infiltration by myeloid cells (such as monocytes and neutrophils) in BDL mice and neuroinflammatory disorders [48, 59]. Given that we have defined microglia as CD45^lo-int^CD11b^+^, a small portion of infiltrating myeloid cells might have contaminated microglial samples, especially in BDL conditions, changing cellular composition across experimental groups. However, it has recently become clear that in pathological conditions, microglia and CNS-infiltrating monocyte-derived macrophages (MDM) exhibit bidirectional plasticity, where microglia can adopt an inflammatory macrophage like phenotype, while infiltrating MDMs can adopt a microglial gene signature. This bidirectional plasticity is however highly time- and pathology-dependent. Indeed, infiltrating MDMs can adopt a microglial signature in animal models of multiple sclerosis [98] and a significant proportion of CD45^hi^ cells express bona fide microglial markers like *Tmem119* and *P2ry12* in models of stroke and cerebrovascular degeneration [99]. Moreover, a recent report showed that in stroke, almost half of the CNS-infiltrating MDMs adopt a CD45^lo^ phenotype [100]. Collectively, these reports indicate that using the gold standard gating strategy defining microglia as CD45^lo-int^CD11b^+^, it is often very difficult to distinguish MDMs and resident microglia, depending on experimental conditions. In future studies, using microglia-specific fluorescent reporter lines like the Cx3CR1-CreERT2:R26-YFP [101] or single-cell sequencing approaches might be used to solve this issue.

Lowering ammonia remains the mainstay of HE treatment [6, 14]. As most of the observed mechanisms (inflammation, hypoxia, oxidative stress, senescence) can be linked to hyperammonemia, it is likely that lowering ammonia will have positive effects on these pathological pathways as well. Our data suggest that continued and renewed efforts should be undertaken to modulate the cerebral (and peripheral) inflammatory response, as these could ameliorate pathological phenotypes in both glial cell types next to lowering ammonia. Despite some recent promising results regarding the anti-inflammatory effects of albumin in minimal HE [102], the knowledge of the importance of inflammation in HE has not led to approved treatments thus far. Additionally, interventions directed specifically at astrocytes (e.g., senescence) or microglia (e.g., inhibiting immune cell infiltration) could be of interest. Finally, given the overlap with other neuroinflammatory disorders like septic encephalopathy, therapeutic strategies might be adapted from these diseases as well.

## Conclusions

In conclusion, glial cells exhibit a sustained transcriptional activation in experimental HE, more pronounced in microglia, and enriched for inflammatory signaling. Innate immune cells are attracted to the BDL mouse brain, likely mainly driven by signals from microglia, but with added roles for astrocytes and putatively other brain cells. Glial cell responses in BDL mice exhibit similarities with other neuroinflammatory disorders, suggesting overlapping pathophysiology. Our dataset identifies key molecular mechanisms involved in experimental HE and provides a valuable resource for the study of glial dysfunction in HE and development of glial-directed treatments.

## Supplementary Information

**Additional file 1: Figure S1.**Gating strategy used for isolation of astrocytes and microglia.FSC-SSC profile of ACSA2^hi^ astrocytes and ACSA2^int^ debris and contaminants.Expression levels of brain cell markers in ACSA2^hi^ and ACSA2^int^ + ACSA2^hi^ compared to RNA isolated from a single cell suspension derived from whole brain.

**Additional file 2: Figure S2.** Mean fluorescence intensity of CD45 in CD45^lo-int^CD11b^+^ microglia. Data are derived from a single experiment with *n* = 6–12/group.

**Additional file 3: Figure S3.** ALT levels of sham and BDL mice used for RNA-Seq. Data derived from a single experiment with *n* = 6–12/group.

**Additional file 4: Figure S4.** Myeloid cell accumulation in the BDL mouse brain.Representative flow cytometry plots indicating gating strategy used to identify CD45^hi^CD11b^+^ myeloid cells and CD45^hi^CD11b^−^ lymphoid cells.Relative abundance of myeloid and lymphoid cells in sham/BDL mouse brains 14 and 28 days after induction. Data are pooled from 2 experiments with *n* = 12–18.

**Additional file 5: Figure S5.** Gating strategy used for immune phenotyping in sham and BDL mouse brains.

**Additional file 6: Table S1.** Antibodies used for flow cytometry.

**Additional file 7: Table S2.** Primer sequences for qPCR.

**Additional file 8: Table S3.** Differentially expressed genes in astrocytes and microglia. List of differentially expressed genes between the BDL and Sham group at 14 days and 28 days in astrocytes and microglia, respectively, as determined by edgeR differential expression analysis. Benjamini–Hochberg correction is used to adjust the *p*-values for multiple testing. The listed DEGs have an adjusted *p*-value smaller than 0.05 and a log_2_-ratio > 1 or < − 1.

**Additional file 9: Table S4.** GO analysis. List of enriched GOs in differential expression gene sets, as determined by GO analysis. Analysis was performed on differential gene expression sets of the differential expression analysis between BDL and sham groups at 14 and 28 days after induction, both for microglia and astrocytes.

**Additional file 10: Table S5.** Upstream regulator analysis. List of identified upstream regulators explaining the observed differential gene expression. Analysis was performed on differential gene expression sets of the differential expression analysis between BDL and sham groups at 14 and 28 days after induction, both for microglia and astrocytes.

**Additional file 11: Table S6.** Antibodies used for confocal microscopy.

## Data Availability

The data generated during the current study are available from the corresponding author upon reasonable request. The raw sequencing data generated in this publication have been deposited in NCBI’s Gene Expression Omnibus and are accessible through GEO under accession number GSE230287.

## Abbreviations

AD: Alzheimer’s disease
ALT: Alanine aminotransferase
BBB: Blood–brain barrier
BDL: Bile duct ligation
BSA: Bovine serum albumin
CCR2: C-C chemokine receptor type 2
CLD: Chronic liver disease
CNS: Central nervous system
CPM: Counts per million
CSF: Cerebrospinal fluid
DAM: Disease-associated microglia
DE: Differential expression
DEG: Differentially expressed gene
EDTA: Ethylenediaminetetraacetic acid
FC: Fold change
FACS: Fluorescence-activated cell sorting
GFAP: Glial fibrillary acidic protein
GFP: Green fluorescent protein
GO: Gene Ontology
GSEA: Gene set enrichment analysis
HBSS: Hanks’ balanced salt solution
HE: Hepatic encephalopathy
IPA: Ingenuity pathway analysis
LPS: Lipopolysaccharide
Ly6G: Lymphocyte antigen 6 complex locus G6D
MCAO: Middle cerebral artery occlusion
MDM: Monocyte-derived macrophage
NES: Normalized enrichment score
PCA: Principal component analysis
PBS: Phosphate-buffered saline
PFA: Paraformaldehyde
RES: Running enrichment score
RNA-Seq: RNA sequencing
RT: Room temperature
SEM: Standard error of the mean
TdT: TdTomato
TNF: Tumor necrosis factor
TPM: Transcripts per million
