# Supplementary figures and images for: Experimental hepatic encephalopathy causes early but sustained glial transcriptional changes
Source: J Neuroinflammation. 2023 May 29;20:130. doi: 10.1186/s12974-023-02814-w (PMC10226265; doi:10.1186/s12974-023-02814-w)

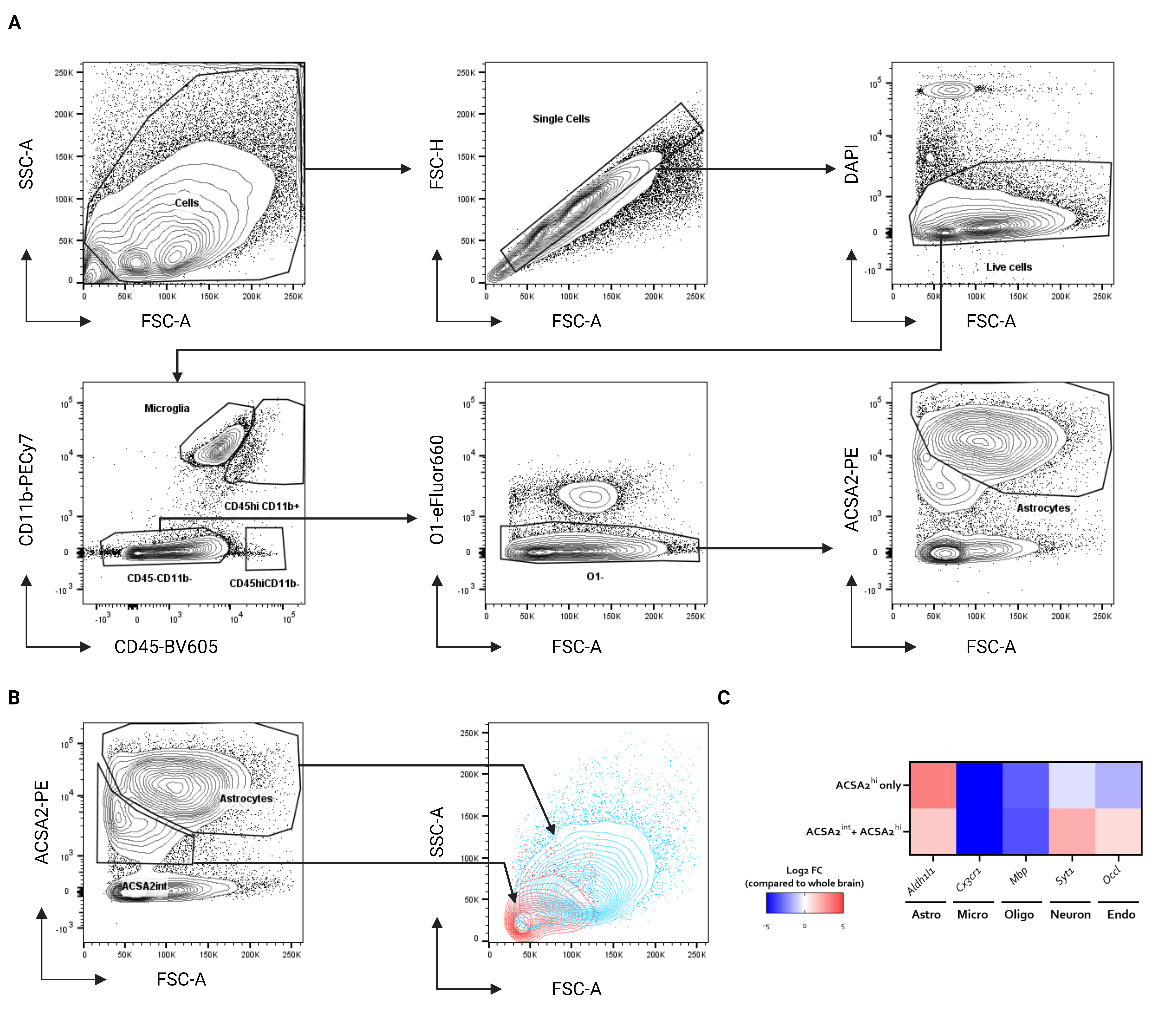

Supplement: Supplementary file 1 — Additional file 1: Figure S1.Gating strategy used for isolation of astrocytes and microglia.FSC-SSC profile of ACSA2hi astrocytes and ACSA2int debris and contaminants.Expression levels of brain cell markers in ACSA2hi and ACSA2int + ACSA2hi compared to RNA isolated from a single cell suspension derived from whole brain. [file 12974_2023_2814_MOESM1_ESM.png]

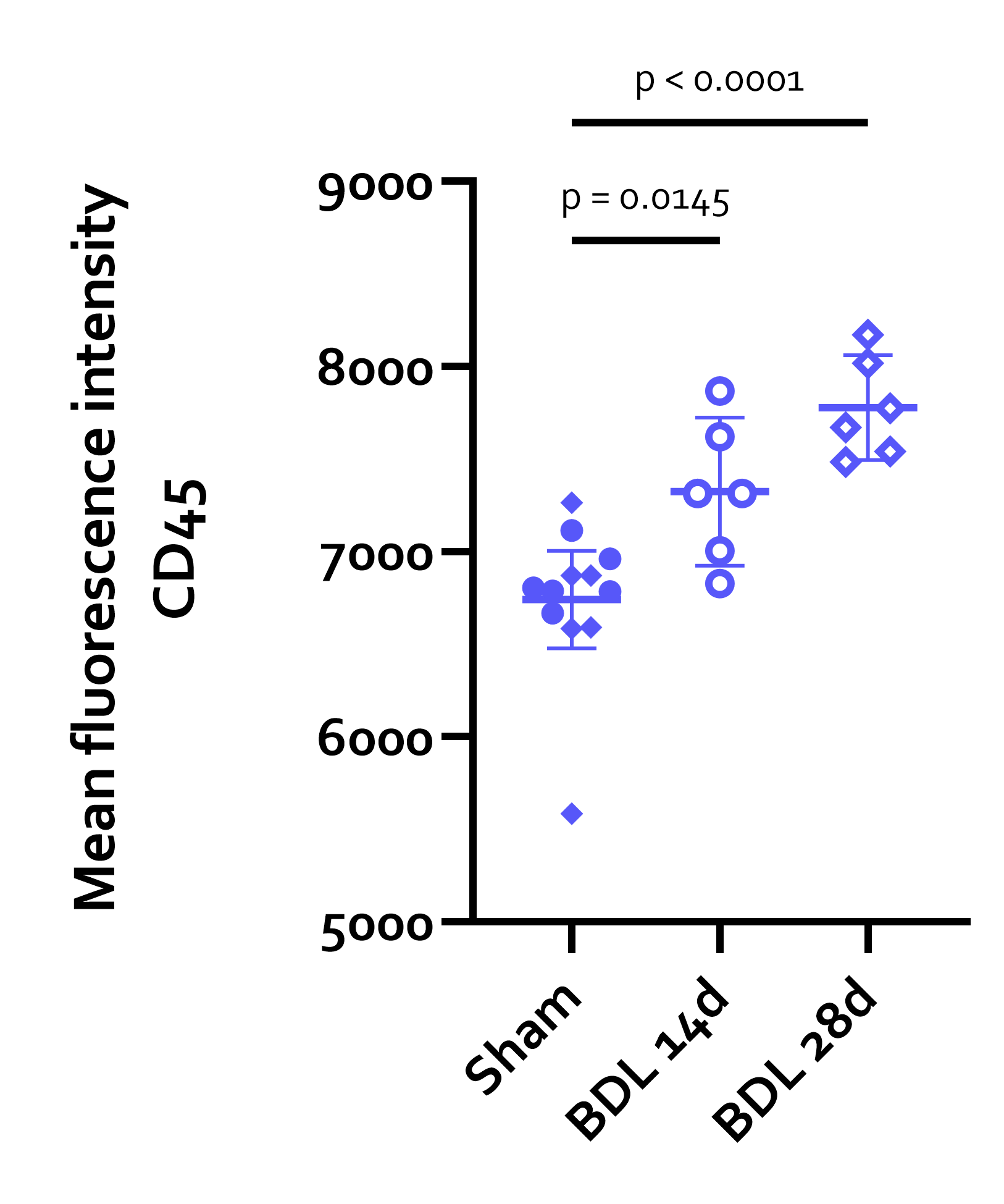

Supplement: Supplementary file 2 — Additional file 2: Figure S2. Mean fluorescence intensity of CD45 in CD45lo-intCD11b+ microglia. Data are derived from a single experiment with n = 6–12/group. [file 12974_2023_2814_MOESM2_ESM.png]

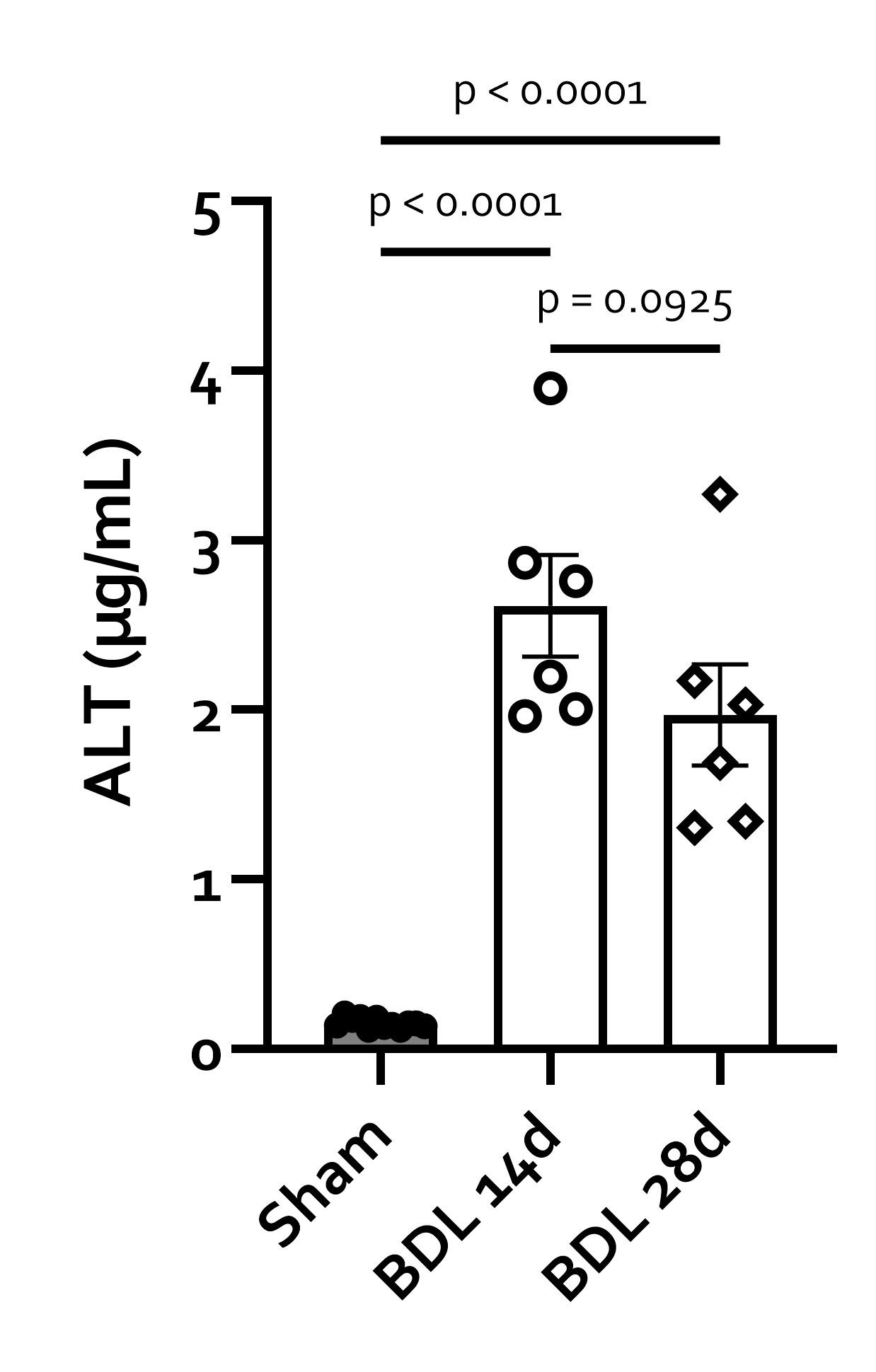

Supplement: Supplementary file 3 — Additional file 3: Figure S3. ALT levels of sham and BDL mice used for RNA-Seq. Data derived from a single experiment with n = 6–12/group. [file 12974_2023_2814_MOESM3_ESM.png]

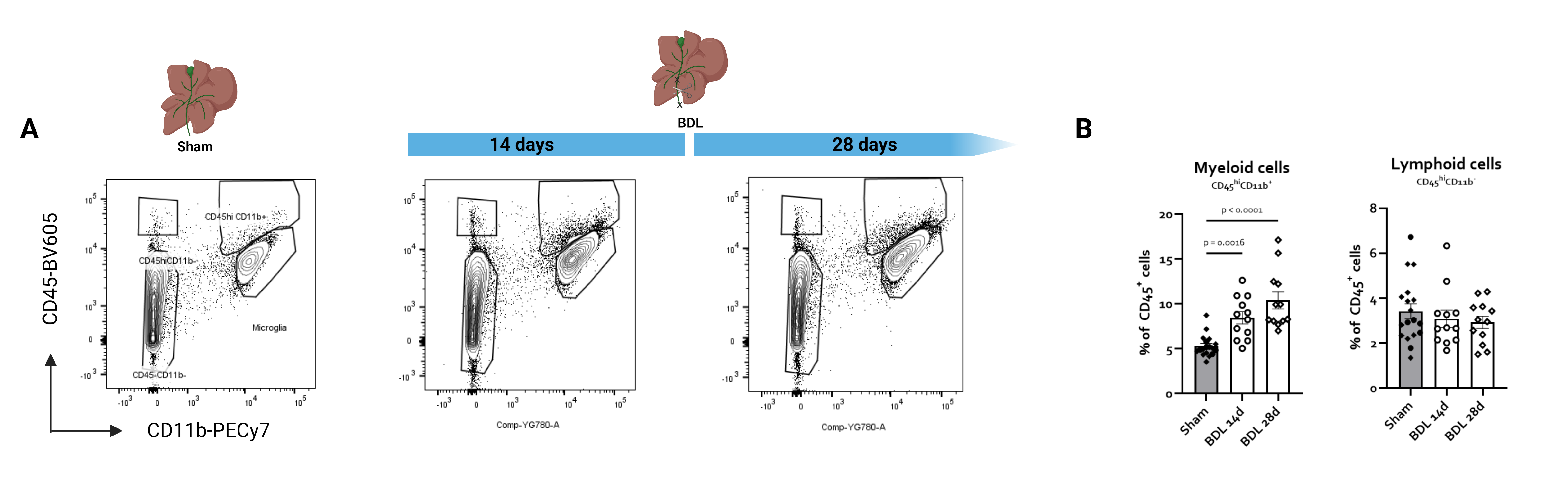

Supplement: Supplementary file 4 — Additional file 4: Figure S4. Myeloid cell accumulation in the BDL mouse brain.Representative flow cytometry plots indicating gating strategy used to identify CD45hiCD11b+ myeloid cells and CD45hiCD11b− lymphoid cells.Relative abundance of myeloid and lymphoid cells in sham/BDL mouse brains 14 and 28 days after induction. Data are pooled from 2 experiments with n = 12–18. [file 12974_2023_2814_MOESM4_ESM.png]

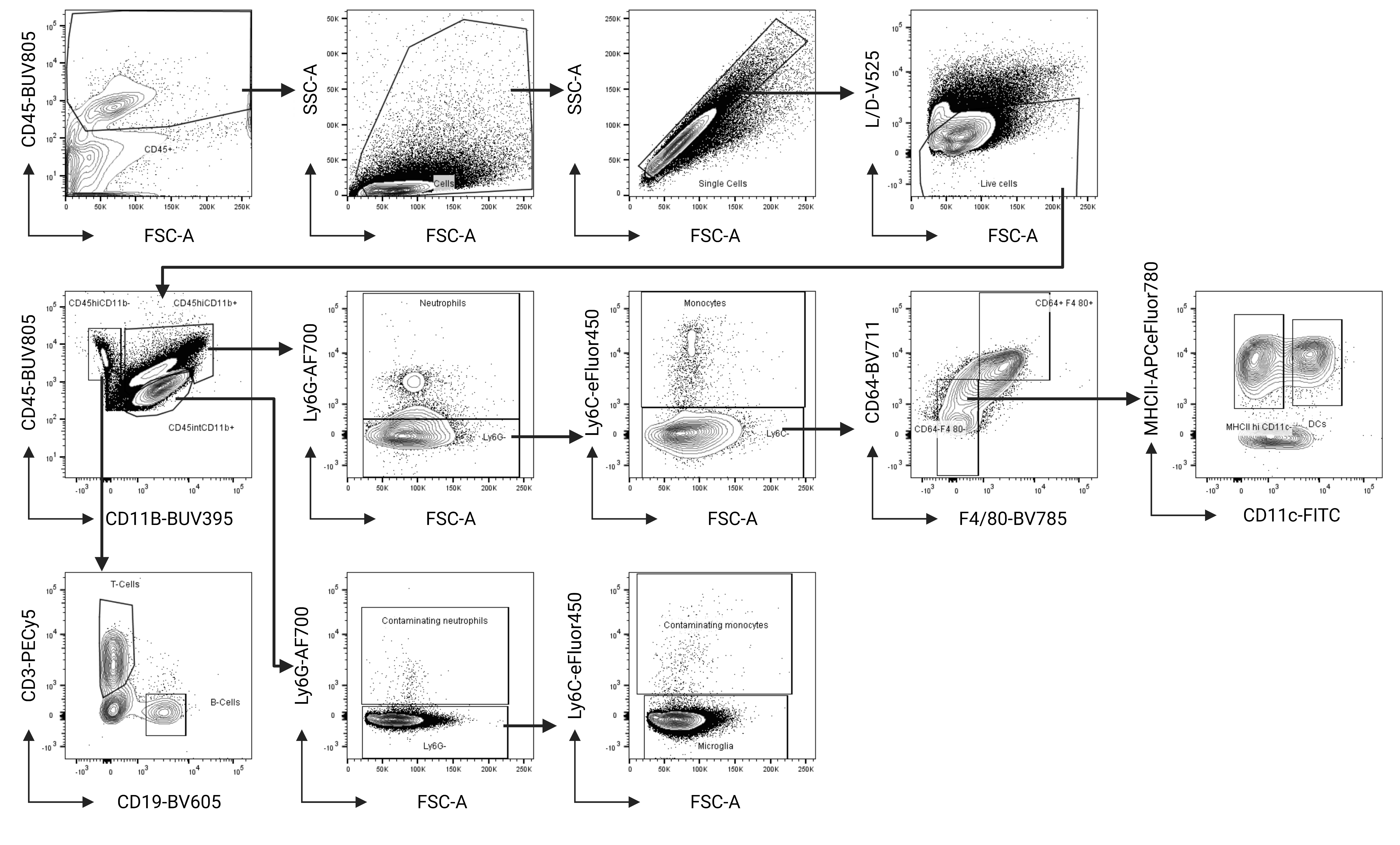

Supplement: Supplementary file 5 — Additional file 5: Figure S5. Gating strategy used for immune phenotyping in sham and BDL mouse brains. [file 12974_2023_2814_MOESM5_ESM.png]
